# Aurora-A drives sorafenib resistance by scaffolding stress granule assembly via phase separation

**DOI:** 10.1073/pnas.2516469123

**Published:** 2026-04-20

**Authors:** Lingyu Kong, Fumei Zhong, Fazhi Yu, Ting Wang, Gang Wang, Yu Bai, Han Xia, Zihang Pan, Mingxue Liu, Yan Zhang, Rui Feng, Jiahai Zhang, Yingying Du, Kaiguang Zhang, Jing Guo, Ke Ruan, Zhenye Yang

**Affiliations:** ^a^Department of Digestive disease, The First Affiliated Hospital of University of Science and Technology of China (USTC), State Key Laboratory of Immune Response and Immunotherapy, Division of Life Sciences and Medicine, University of Science and Technology of China, Hefei 230027, China; ^b^Ministry of Education (MOE) Key Laboratory for Cellular Dynamics and Membraneless Organelle, Division of Life Sciences and Medicine, University of Science and Technology of China, Hefei 230027, China; ^c^Chinese Academy of Sciences Key Laboratory of Regenerative Biology, Joint School of Life Sciences, Guangzhou Institutes of Biomedicine and Health, Chinese Academy of Sciences, Guangzhou Medical University, Guangzhou 511436, China; ^d^Department of Oncology, the First Affiliated Hospital of Anhui Medical University, Hefei 230022, China; ^e^Center for Advanced Interdisciplinary Science and Biomedicine, Institute of Health and Medical Technology, Division of Life Sciences and Medicine, University of Science and Technology of China, Hefei 230026, China; ^f^Anhui Key Laboratory of Molecular Oncology, Division of Life Sciences and Medicine, Institute of Cancer Research, University of Science and Technology of China, Hefei 230026, China

**Keywords:** Aurora-A, stress granule, sorafenib, phase separation, tumor therapy

## Abstract

The mitotic kinase Aurora-A is frequently overexpressed in cancers and contributes to tumor progression and therapy resistance, yet the mechanisms underlying its role in drug resistance remain unclear. Here, we show that sorafenib treatment triggers Aurora-A phase separation, leading to its recruitment into stress granules (SGs), membraneless organelles that promote cancer cell survival. Aurora-A facilitates robust SGs assembly, thereby conferring sorafenib resistance. Mechanistically, upon sorafenib-induced SGs formation, Aurora-A binds RNA via positively charged lysine/arginine residues within its intrinsically disordered region (IDR). Mutating these K/R residues with IDR of Aurora-A disrupts its RNA binding, impairs SGs assembly, and resensitizes cancer cells to sorafenib. Together, our work identifies Aurora-A as a kinase-activity-independent, RNA-binding scaffold essential for stress-adaptive biomolecular condensation, revealing a druggable phase-separation axis distinct from canonical Aurora-A kinase signaling.

## Significance

This study reveals how cancer cells resist the targeted drug sorafenib. We found that the protein Aurora-A, known for its role in cell division, can form liquid-like droplets in the cytoplasm under drug stress. These droplets help build protective structures called stress granules, which shield cancer cells from the drug’s lethal effects. Importantly, this process does not require Aurora-A’s enzymatic activity but depends on its ability to bind RNA. By blocking this droplet-forming ability, we successfully made tumors more sensitive to sorafenib in mice. Our work uncovers a kinase-independent mechanism of drug resistance and highlights a promising target for overcoming treatment resistance in cancer.

Aurora kinase A (Aurora-A/AURKA) is a serine/threonine kinase essential for mitotic progression and a well-established oncogenic driver, primarily through its kinase-dependent functions (1–4). Its overexpression is strongly associated with tumorigenesis and therapy resistance across diverse cancers (5–10). Notably, combined treatment with Aurora-A inhibitors and targeted therapies markedly suppresses tumor growth, highlighting Aurora-A as an attractive therapeutic target (11–19). However, the precise mechanisms by which Aurora-A drives resistance to various targeted agents remain incompletely understood.

Recent studies reveal that Aurora-A can undergo liquid–liquid phase separation (LLPS) in vitro. During mitosis, its N-terminal intrinsically disordered region (IDR) mediate interactions with its own C-terminal domain and with BuGZ, promoting Aurora-A condensation at centrosomes, its T288 phosphorylation, and subsequent centrosome maturation (20, 21). This suggests LLPS regulates Aurora-A’s mitotic functions. It remains unknown whether the aberrant overexpression of Aurora-A in cancer cells triggers LLPS under therapeutic stress and whether this process contributes to drug resistance.

Stress granules (SGs) are dynamic, membraneless condensates of RNA and RNA-binding proteins (e.g., G3BP1/2) that assemble under diverse cellular stresses (22, 23). Compelling evidence links SGs formation to resistance against chemotherapy and targeted therapy-induced apoptosis (24–26). Multiple antitumor therapeutics, such as sorafenib, trigger eIF2α-dependent SGs assembly, thereby enabling cancer cells to survive therapeutic stress (27–30). Sorafenib-mediated endoplasmic reticulum stress activates PERK-eIF2α signaling to promote SGs formation, establishing a survival mechanism in treated malignancies (30). However, whether SGs assembly is a direct driver of therapeutic resistance and the underlying mechanisms require further elucidation. Although both Aurora-A and SGs have independently been linked to therapy resistance, a potential functional connection, particularly one mediated by phase separation, has not been explored.

Here, we report that sorafenib induces pronounced cytoplasmic condensation of Aurora-A into SGs. Mechanistically, the IDR of Aurora-A directly binds RNA via conserved lysine and arginine residues, forming an Aurora-A–RNA–G3BP1 ternary complex independently of its kinase activity to drive SGs assembly. In vivo, tumors expressing a phase-separation-deficient Aurora-A mutant show heightened sensitivity to sorafenib. Our work unveils a kinase-independent, phase-separation-dependent role for cytoplasmic Aurora-A in promoting SG-mediated therapy resistance.

## Result

### Sorafenib Triggers Aurora-A Phase Separation and Colocalization with SGs.

**Fig. 1.**
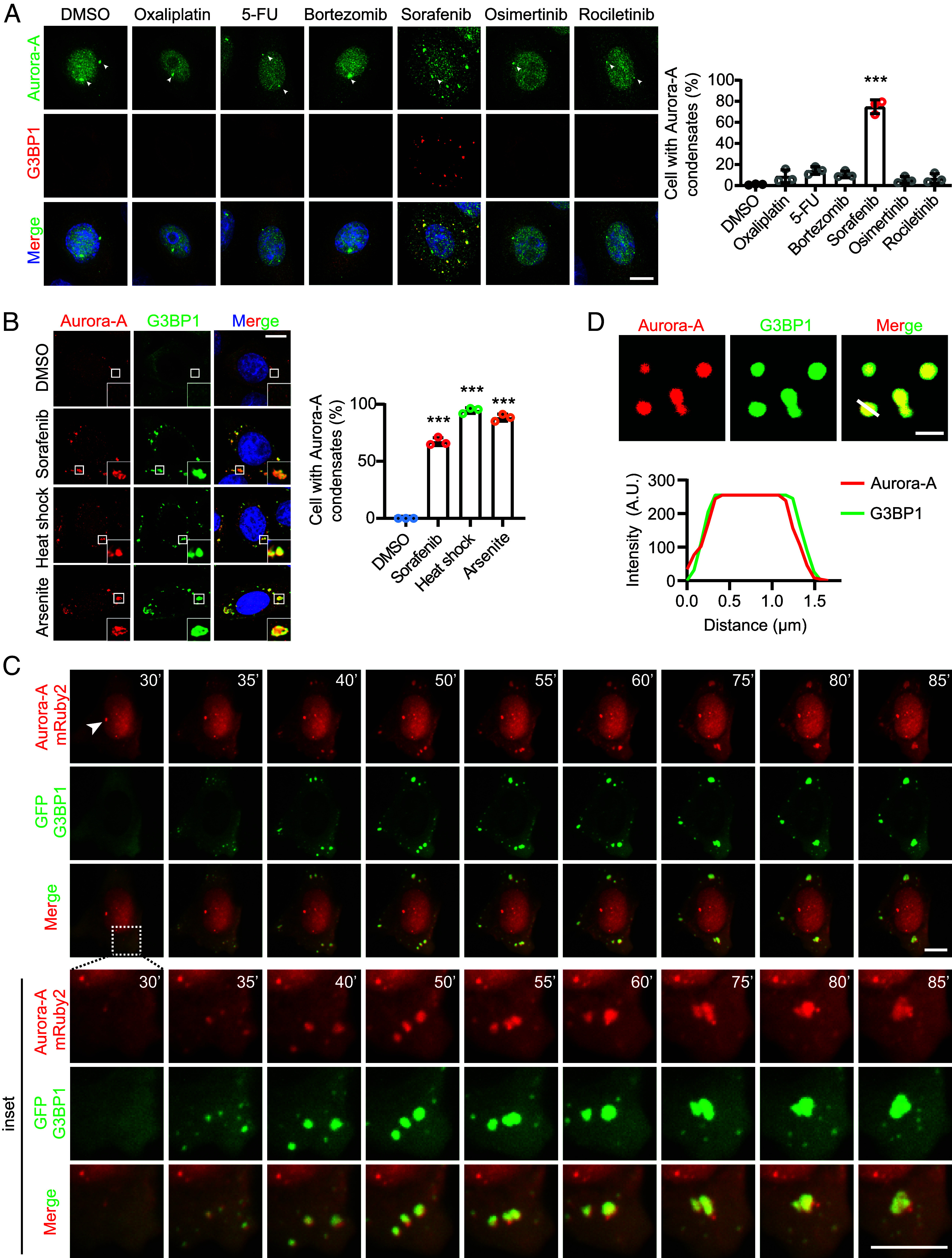
Sorafenib triggers Aurora-A phase separation and colocalization with SGs. (*A*) Immunofluorescence analysis of protein localization for Aurora-A (green) and G3BP1 (red) in HeLa cells that were treated with dimethyl sulfoxide (DMSO), 100 μM sorafenib, 200 μM oxaliplatin, 100 μM 5-FU, 10 μM bortezomib, 10 μM osimertinib, and 10 μM rociletinib for 2 h each. The nucleus was stained with DAPI (blue). The arrowhead indicates the centrosome with no change. (Scale bar, 10 μm.) Quantification of the proportion of cells with Aurora-A granule formation after different drug treatments. n = 3 biologically independent experiments. Mean ± SD, ***P < 0.001. (*B*) Protein localization assessed by immunofluorescence analysis for Aurora-A (red) and G3BP1 (green) in HeLa cells that were treated with DMSO, 100 μM sorafenib (2 h), 100 μM arsenite (1 h), and heat shock at 42 °C (30 min). The nucleus was stained with DAPI (blue). Quantification of the proportion of cells with Aurora-A granule formation following treatment with individual drugs. n = 3 biologically independent experiments. Mean ± SD, ***P < 0.001. (*C*) Time-lapse microscopy of Aurora-A-mRuby2 and GFP-G3BP1 granule dynamics with 100 μM sorafenib treated. The arrowhead indicates the centrosome with no change. (Scale bar, 10 μm.) Magnification of the demarcated region was shown as the *Inset* for each condition from the merged images. (*Inset* scale bar, 10 μm.) (*D*) LLPS was reconstituted in vitro using purified recombinant proteins. The assay mixture contained 1 μM Alexa Fluor™ 594-labeled Aurora-A(red) and 20 μM Alexa Fluor™ 488-labeled G3BP1 (green). To initiate cophase separation, 50 ng/μL total RNA was added as a scaffolding molecule. Images show representative data from three independent experiments. (Scale bar, 2 μm.) Profile intensity showing Aurora-A/G3BP1 fluorescence signals of the white lines for each condition from the merged images.

To investigate whether Aurora-A undergoes phase separation under therapeutic stress, we treated HeLa cells expressing Aurora-A–mRuby2 with various anticancer agents, including oxaliplatin, 5-fluorouracil (5-FU), bortezomib, sorafenib, and the third-generation EGFR tyrosine kinase inhibitors osimertinib and rociletinib (11, 15, 17, 31, 32). Aurora-A localization was monitored from 30 min to 5 h after treatment. Among all agents tested, only sorafenib robustly induced cytoplasmic condensation of Aurora-A in a dose-dependent manner (Fig. 1*A* and *SI Appendix*, Fig. S1*A*). In contrast, Aurora-A remained predominantly centrosomal (arrowhead, Fig. 1*A*) without obvious condensation after treatment with the other drugs (Fig. 1*A* and *SI Appendix*, Fig. S1*A*).

Because SGs assemble in response to chemotherapeutic stress and promote cell survival (33–35), we asked whether sorafenib-induced Aurora-A condensates associate with SGs. Immunofluorescence analysis showed that these condensates co-localized with the SG markers G3BP1 (Fig. 1*A*) and TIA1 (*SI Appendix*, Fig. S1*B*) (22). Furthermore, under canonical SG-inducing conditions such as sodium arsenite treatment and heat shock (36), Aurora-A was similarly recruited into G3BP1-positive granules (Fig. 1*B*). Live-cell imaging of HeLa cells co-expressing Aurora-A–mRuby2 and EGFP-G3BP1 revealed that sorafenib treatment led to synchronized nucleation, fusion, and dissolution of dual-positive droplets containing both Aurora-A and G3BP1, while centrosomal Aurora-A (arrowhead, Fig. 1*C*) remained unchanged (Fig. 1*C* and *SI Appendix*, Fig. S1*C*). In vitro reconstitution assays further demonstrated that purified Aurora-A and G3BP1 co-condense into dynamic hybrid droplets (Fig. 1*D*).

Given the clinical use of sorafenib in hepatocellular carcinoma (37), we next examined whether sorafenib also induces Aurora-A phase separation and SGs localization in liver cancer cell lines Hep-3B and PLC/PRF/5. Consistent with the observations in HeLa cells, sorafenib treatment triggered the formation of Aurora-A-positive droplets that co-localized with SGs in both liver cancer lines (*SI Appendix*, Fig. S1*D*). Together, these data demonstrate that sorafenib specifically induces Aurora-A phase separation and its recruitment into SGs.

### Aurora-A Drives SGs Assembly through Its Phase Separation during Sorafenib-Induced Stress.

**Fig. 2.**
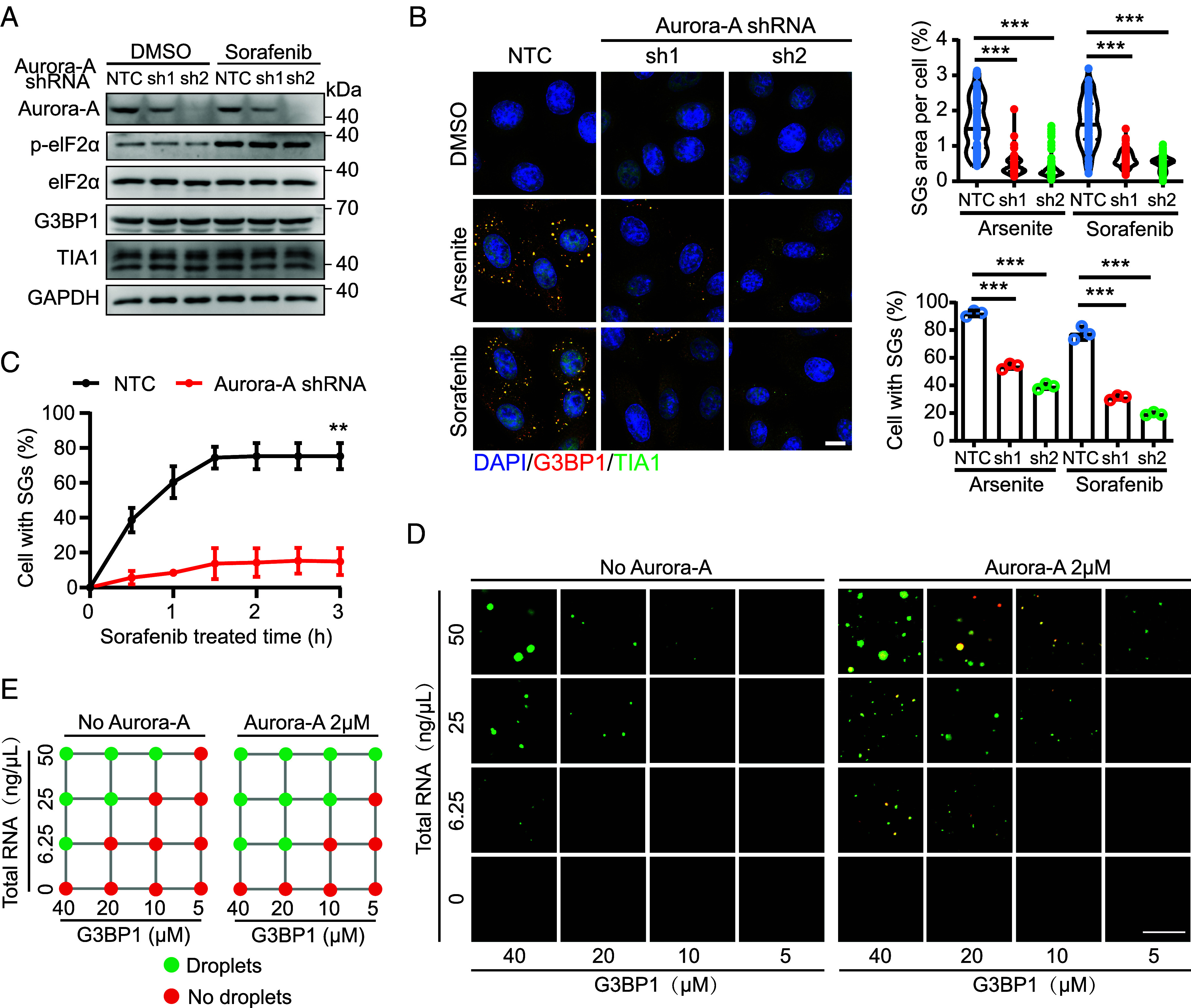
The phase separation of Aurora-A drives SGs formation under sorafenib. (*A*) Western blotting showing the level changes of endogenous Aurora-A in HeLa cells upon Aurora-A knock down with shRNA. Knockdown of Aurora-A has no effect on the expression level of SGs core proteins (G3BP1, TIA1). (*B*) Confocal imaging of nontarget control (NTC) and Aurora-A shRNA1, shRNA2 treated with 100 μM arsenite (1 h), and 100 μM sorafenib (2 h) to visualize the formation of SGs. Quantification of the proportion of cells with SGs formation (n = 3) and the area of SGs per cell (n = 100). Mean ± SD, ***P < 0.001. (*C*) Quantification of the proportion of Aurora-A shRNA-mRuby2 cells forming SGs is provided. n = 3 biologically independent experiments. Mean ± SD, **P < 0.01. (*D*) Confocal imaging of the effect of additional Alexa Fluor™ 594-labeled Aurora-A(red) on the phase separation of Alexa Fluor™ 488-labeled G3BP1 (green). All proteins used in this assay were purified in vitro. To initiate cophase separation, total RNA was added as a scaffolding molecule. Images show representative data from three independent experiments. (Scale bar, 10 μm.) (*E*) Summary of phase separation behaviors of G3BP1 in *D*. No droplet (red); formation droplets (green).

To determine whether Aurora-A functionally contributes to SGs assembly, we depleted Aurora-A using shRNA (Fig. 2*A*). Aurora-A knockdown significantly reduced both the percentage of cells containing SGs and the total SGs area per cell following induction by sorafenib or arsenite (Fig. 2*B*). Notably, Aurora-A depletion did not alter the expression of core SG proteins or affect sorafenib-induced phosphorylation of eIF2α (Fig. 2*A*), suggesting that Aurora-A participates in the assembly process rather than upstream signaling events that initiate SGs formation. Live-cell imaging further demonstrated that loss of Aurora-A abolished the time-dependent accumulation of SGs during sorafenib treatment (Fig. 2*C* and *SI Appendix*, Fig. S2*A*).

In vitro reconstitution assays showed that G3BP1 formed RNA-dependent condensates, which were markedly enhanced upon addition of Aurora-A (Fig. 2 *D* and *E*), supporting a direct role for Aurora-A in promoting SGs formation. In contrast, sorafenib alone did not enhance droplet formation in this cell-free system (*SI Appendix*, Fig. S2*B*), indicating that sorafenib does not directly stimulate Aurora-A phase separation or SGs assembly in vitro.

To examine whether Aurora-A phase separation depends on SGs nucleation, we generated G3BP1/2 double-knockout (DKO) HeLa cells (22). In the absence of SGs formation, neither sorafenib nor arsenite induced condensation of Aurora-A (*SI Appendix*, Fig. S2 *C* and *D*). Similarly, cycloheximide treatment, which prevents SGs assembly by trapping mRNAs in polysomes (38), also abolished Aurora-A condensation (*SI Appendix*, Fig. S2*E*). SGs are composed of translationally arrested mRNAs, small ribosomal subunits, and RNA-binding proteins such as G3BP1 (39). Polysome profiling revealed that sorafenib-induced translational arrest caused both Aurora-A and G3BP1 to cofractionate with the 40S ribosomal subunit, whereas inhibition of SGs assembly by cycloheximide reduced their association with the 40S fraction (*SI Appendix*, Fig. S2*F*). Together, these data suggest that Aurora-A may engage in SGs assembly by interacting with G3BP1 and the pool of translationally stalled mRNA–40S complexes generated upon sorafenib treatment.

In summary, these findings demonstrate that Aurora-A promotes SGs assembly and that its phase separation is tightly linked to SGs formation.

### Aurora-A Scaffolds SGs Assembly Dispensing with Its Kinase Functionality.

**Fig. 3.**
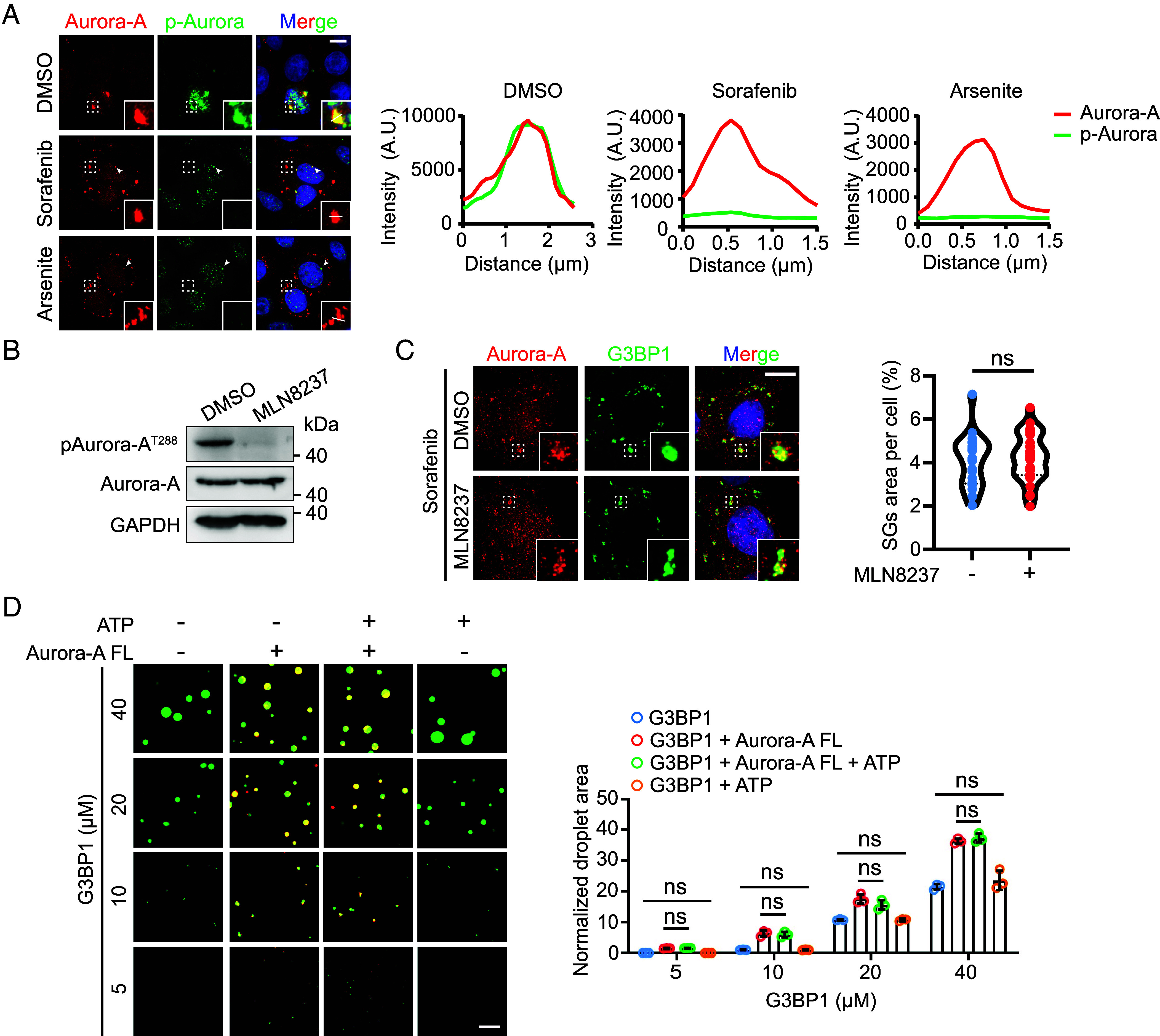
Aurora-A scaffolds SGs assembly dispensing with its kinase functionality. (*A*) Protein localization assessed by immunofluorescence analysis for Aurora-A and p-Aurora in HeLa cells that were treated with DMSO, 100 μM sorafenib (2 h), 100 μM arsenite (1 h). Arrowheads indicate the normal centrosomal accumulation of p-Aurora. (Scale bar, 10 μm.) Profile intensity showing Aurora-A/p-Aurora fluorescence signals of the white lines for each condition from the merged images. (*B*) Western blotting showing the level changes of pAurora-A T288 in HeLa cells upon 100 nM MLN8237 treated. (*C*) Immunofluorescence analysis for Aurora-A phase separation combined with MLN8237 and sorafenib. (Scale bar, 10 μm.) Quantification of the area of SGs per cell. n = 30, Mean ± SD, ns, nonsignificance. (*D*) In vitro kinase assays analyzed the impact of Aurora-A enzymatic activity on G3BP1 phase separation. Confocal microscopy images show the coexistence of Alexa Fluor™ 488-labeled G3BP1 (green) and Alexa Fluor™ 594-labeled Aurora-A (red). The experiment was performed using purified proteins, with the addition of 1 μM Aurora-A, and 200 μM ATP was either included or excluded as needed. Images show representative data from three independent experiments. (Scale bar, 10 μm.) Quantification of the area of G3BP1 droplets. Mean ± SD, ns, nonsignificance.

We next asked whether Aurora-A kinase activity is required for SGs assembly. While sorafenib and arsenite did not alter total Aurora-A protein levels, only arsenite increased its phosphorylation at the activation loop (*SI Appendix*, Fig. S3*A*). Notably, both stimuli induced robust Aurora-A condensation, suggesting that phosphorylation is dispensable for phase separation. Consistent with this, Aurora-A within stress-induced condensates showed no detectable phosphorylation, in contrast to its phosphorylated state at centrosomes (arrowhead, Fig. 3*A*) or spindle poles (*SI Appendix*, Fig. S3*B*). Pharmacological inhibition of Aurora-A kinase activity with MLN8237 did not impair Aurora-A phase separation, its recruitment to SGs, or condensate dynamics (Fig. 3 *B* and *C* and *SI Appendix*, Fig. S3*C*). Similarly, a kinase-dead Aurora-A mutant (D274A) (40) retained full capacity for phase separation and SGs localization (*SI Appendix*, Fig. S3*D*). Using an in vitro reconstitution assay, we further confirmed that enhancing Aurora-A enzymatic activity with ATP had no effect on G3BP1 droplet formation (Fig. 3*D*). Together, these results demonstrate that Aurora-A phase separation and its role in SGs assembly are independent of its kinase function.

Given that BuGZ promotes Aurora-A condensation at spindle poles during mitosis (20, 21), we tested whether it also scaffolds Aurora-A under stress. BuGZ did not colocalize with cytoplasmic Aurora-A condensates and remained predominantly nuclear (*SI Appendix*, Fig. S3*E*), ruling out its involvement in stress-induced Aurora-A phase separation.

### Formation of Aurora-A-RNA-G3BP1 Condensates Is Mediated by an Interaction between Aurora-A’s IDR and RNA.

**Fig. 4.**
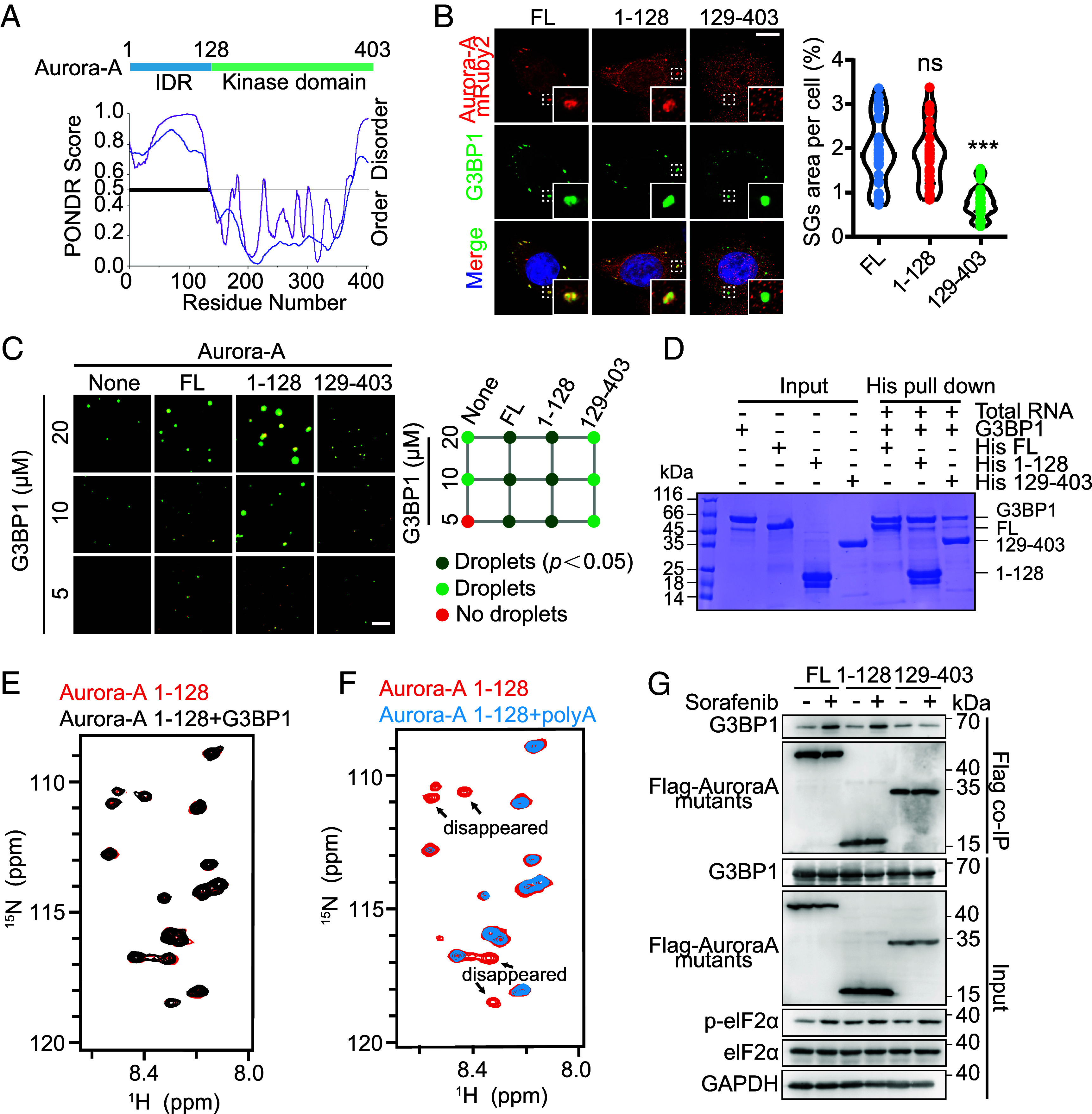
Formation of Aurora-A-RNA-G3BP1 condensates is mediated by an interaction between Aurora-A’s IDR and RNA. (*A*) Sequence features of Aurora-A predicted by PONDOR. The line at 0.5 (y axis) is the cutoff for the disorder (>0.5) and order (<0.5) predictions. VLS2 (purple), VL3 (blue) are predictors for disordered dispositions. (*B*) Protein localization assessed by immunofluorescence analysis for Aurora-A truncated mutants (FL, 1-128, 129-403) and G3BP1 in HeLa cells that were treated with 100 μM sorafenib (2 h). (Scale bar, 10 μm.) Quantification of the area of SGs per cell. n = 20, Mean ± SD, ***P < 0.001; ns, nonsignificance. (*C*) LLPS of purified G3BP1 at different concentrations with 50 ng/μL total RNA, with 1 μM Aurora-A FL, 1-128, or 129-403 respectively. Images show representative data from three independent experiments. (Scale bar, 10 μm.) No droplet (red); formation droplets (green); according to the quantification of the area of G3BP1 droplets in *SI Appendix*, Fig. S4*B* compared to none *P* < 0.05 (deep green). (*D*) His pull-down assay demonstrated RNA-dependent interaction between G3BP1 and the N-terminal domain of Aurora-A. Purified His-tagged Aurora-A FL, 1-128, 129-403 (200 μg) were incubated with untagged G3BP1 (200 μg) and total RNA (200 μg). Proteins bound to Ni-NTA beads were analyzed by Coomassie blue staining. (*E*) Overlay of ^1^H-^15^N-HSQC spectra of either 100 μM ^15^N-labeled Aurora-A 1-128 alone (red) or in complex with a 1:1 ratio of unlabeled G3BP1 (black). (*F*) Overlay of ^1^H-^15^N-HSQC spectra of either 100 μM Aurora-A 1-128 alone (red) or in complex with a 1:1 ratio of unlabeled 25nt-polyA (blue). Arrows indicate residues that undergo substantial chemical shift perturbations or signal disappearance upon RNA binding. (*G*) The sorafenib-induced interaction between Flag-tagged Aurora-A FL/1-128 and G3BP1 was confirmed by Flag co-IP. The interaction between Flag-tagged Aurora-A 129-403 and G3BP1 was reduced in HeLa cells.

Bioinformatic analysis using PONDR identified an N-terminal IDR, 1 to 128, in Aurora-A, which is distinct from its structured kinase domain residues 129 to 403 (Fig. 4*A*). While the kinase domain is well characterized, the role of the Aurora-A IDR in oncogenesis remains unclear. Given recent reports of IDR-mediated phase separation at spindle poles (21), we hypothesized that this region drives stress-induced condensate formation within SGs.

We constructed mRuby2-tagged truncation mutants of Aurora-A, encompassing the IDR (1 to 128) or the kinase domain (129 to 403), and expressed them in HeLa cells. Under sorafenib treatment, the isolated IDR underwent phase separation and localized to SGs, whereas the kinase domain remained diffuse and did not support SGs assembly (Fig. 4*B*). This indicates that the IDR mediates Aurora-A recruitment into SGs and promotes SG formation under stress.

To further validate this, we purified full-length (FL) Aurora-A and the truncation mutants (1 to 128 and 129 to 403) for in vitro assays. The IDR exhibited stronger phase-separation capacity than FL Aurora-A and markedly enhanced G3BP1 droplet formation, while the kinase domain had no effect (Fig. 4*C* and *SI Appendix*, Fig. S4 *A* and *B*). His-pull-down assays confirmed a robust, RNA-dependent interaction between the His-tagged Aurora-A IDR and G3BP1 (Fig. 4*D* and *SI Appendix*, Fig. S4*C*). Because RNA is essential for G3BP1 phase separation and primarily binds its RRM-RGG domain (22), we tested whether Aurora-A 1 to 128 forms a ternary complex with this domain. Indeed, the interaction required RNA (*SI Appendix*, Fig. S4*C*), and dose-dependent pull-down showed saturable binding of Aurora-A 1 to 128 to G3BP1 (*SI Appendix*, Fig. S4*D*).

To examine direct molecular interactions, we performed NMR spectroscopy using ^15^N-labeled Aurora-A 1 to 128. ^1^H-^15^N heteronuclear single quantum coherence (HSQC) spectra revealed no chemical-shift perturbations upon addition of G3BP1 alone, confirming the absence of direct protein-protein binding (Fig. 4*E* and *SI Appendix*, Fig. S4*E*). In contrast, Aurora-A 1 to 128 showed significant binding to RNA (poly-U and poly-A), as evidenced by substantial peak shifts and disappearance of some peaks (Fig. 4*F* and *SI Appendix*, Fig. S4 *F* and *G*). We next validated these interactions in cells using Flag co-immunoprecipitation. Under sorafenib treatment, Flag-tagged FL Aurora-A and the 1 to 128 mutant, but not the kinase domain (129 to 403), co-precipitated with G3BP1 (Fig. 4*G*). Together, these data demonstrate that Aurora-A directly binds RNA via its IDR and thereby integrates into G3BP1–RNA condensates.

Although Aurora-B has been reported to condense in arsenite-induced SGs (41) and shares kinase domain homology with Aurora-A (*SI Appendix*, Fig. S4*H*), we observed distinct behaviors. In both HeLa and U251 cells, sorafenib or arsenite induced robust Aurora-A recruitment to SGs, whereas Aurora-B displayed only weak condensation (*SI Appendix*, Fig. S4 *I* and *L*). Notably, 100 μM sorafenib did not trigger SGs formation in U251 cells under these conditions. The differential condensation of Aurora-A and Aurora-B did not correlate with their basal expression levels (*SI Appendix*, Fig. S4 *J* and *M*). Moreover, Aurora-B knockdown did not impair SGs formation (*SI Appendix*, Fig. S4 *K* and *N*), indicating that Aurora-B is not required for SGs assembly under the stress conditions tested.

### Mutation of Conserved IDR Lysine/Arginine Residues Disrupts Aurora-A-RNA-G3BP1 Condensate.

**Fig. 5.**
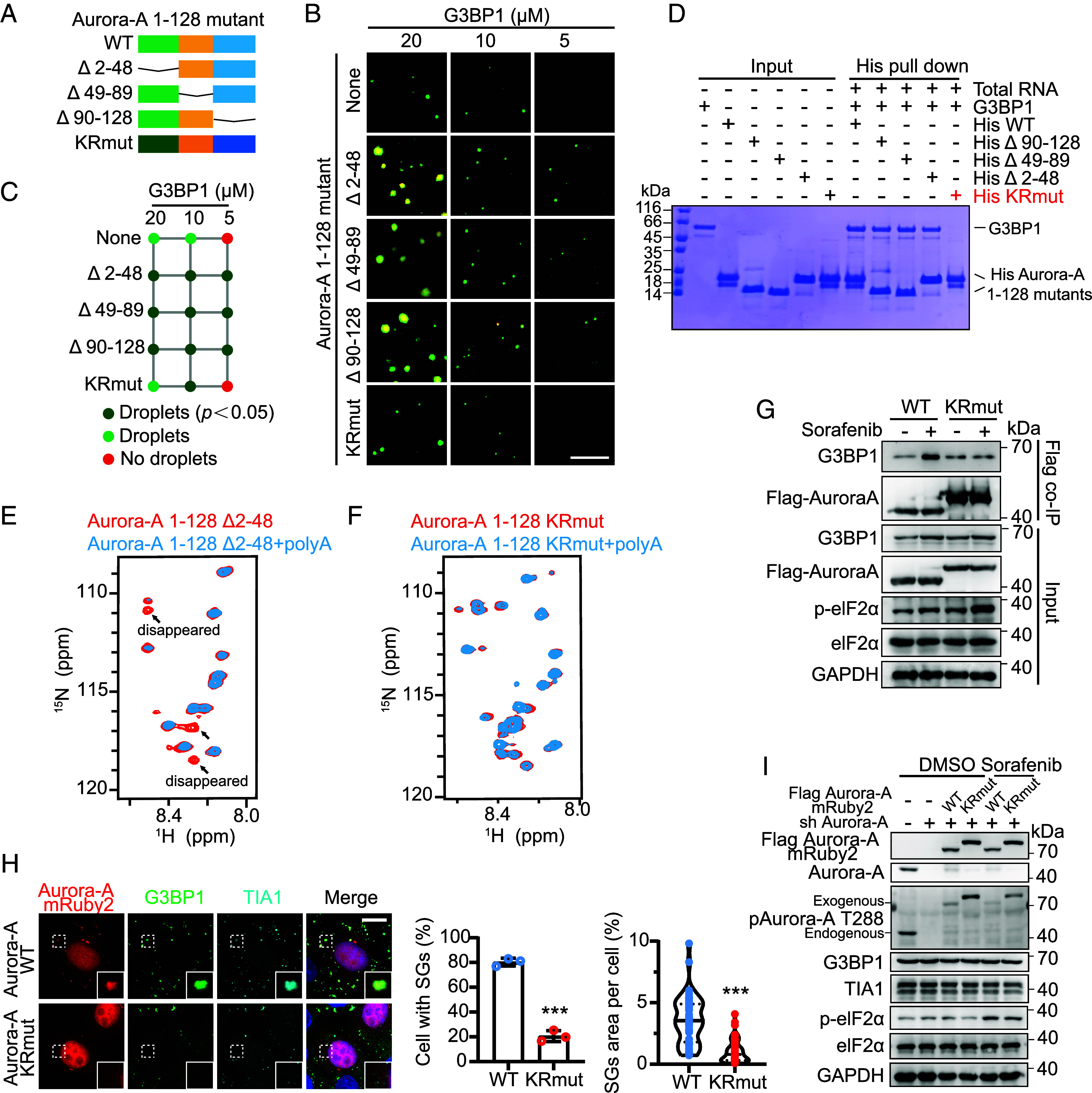
Mutation of conserved IDR lysine/arginine residues disrupts Aurora-A-RNA-G3BP1 condensate. (*A*) Constructs used to investigate the function of individual truncated mutants of Aurora-A 1-128. (*B*) LLPS of purified G3BP1 at different concentrations with 50 ng/μL total RNA, with 1 μM Aurora-A IDR mutants or truncations respectively. Images show representative data from three independent experiments. (Scale bar, 10 μm.) (*C*) Summary of phase separation behaviors of G3BP1 in *B*. No droplet (red); formation droplets (green); according to the quantification of the area of G3BP1 droplets in *SI Appendix*, Fig. S5*E* compared to none *P* < 0.05 (deep green). (*D*) His pull-down assay demonstrated RNA-dependent interaction between G3BP1 and the K/R residues of Aurora-A 1-128. Purified His-tagged Aurora-A IDR mutants or truncations (200 μg) were incubated with untagged G3BP1 (200 μg) and total RNA (200 μg). Proteins bound to Ni-NTA beads were analyzed by Coomassie blue staining. (*E*) Overlay of ^1^H-^15^N-HSQC spectra of either 100 μM ^15^N-labeled Aurora-A 1-128 Δ2-48 alone (red) or in complex with a 1:1 ratio of unlabeled 25nt-polyA (blue). Arrows indicate residues that undergo substantial chemical shift perturbations or signal disappearance upon RNA binding. (*F*) Overlay of ^1^H-^15^N-HSQC spectra of either 100 μM ^15^N-labeled Aurora-A 1-128 KRmut alone (red) or in complex with a 1:1 ratio of unlabeled 25nt-polyA (blue). (*G*) The sorafenib-induced interaction between Flag-tagged Aurora-A WT and G3BP1 was confirmed by co-IP. The interaction between Flag-tagged Aurora-A KRmut and G3BP1 was reduced in HeLa cells. (*H*) Confocal imaging of mRuby2-tagged Aurora-A-WT and Aurora-A-KRmut treated with 100 μM sorafenib (2 h) to visualize the formation of SGs in HeLa cells. (Scale bar, 10 μm.) Quantification of the proportion of cells with SGs formation (n = 3) and the area of SGs per cell (n = 50), Mean ± SD, ***P < 0.001. (*I*) Validation of Flag-tagged FL Aurora-A wild type mRuby2 (Aurora-A-WT), Aurora-A K/R residues mutant mRuby2 (Aurora-A-KRmut) overexpression and endogenous Aurora-A KD with shRNA in HeLa cells. The KRmut did not affect Aurora-A T288 phosphorylation, nor the expression of SG core proteins G3BP1 and TIA1, or eIF2α phosphorylation.

Sequence alignment revealed minimal homology between the N-terminal regions of Aurora-A and Aurora-B (*SI Appendix*, Fig. S4*H*). Given this divergence, we subdivided the Aurora-A N-terminal IDR into three segments (residues 2 to 48, 49 to 89, and 90 to 128) for functional analysis (Fig. 5*A*). Because protein–RNA interactions are largely driven by electrostatics (42), and the Aurora-A IDR is highly enriched in positively charged lysine (K) and arginine (R) residues (*SI Appendix*, Fig. S5 *A* and *B*), we generated a charge-reversal mutant (KRmut) in which all K/R residues within the IDR were replaced with negatively charged aspartate (D) or glutamate (E) (Fig. 5*A* and *SI Appendix*, Fig. S5*C*).

We first purified the Aurora-A 1 to 128 truncation variants (Δ2 to 48, Δ49 to 89, Δ90 to 128, and KRmut) and examined their phase-separation behavior (*SI Appendix*, Fig. S5*D*) and ability to promote G3BP1 condensation (Fig. 5 *B* and *C* and *SI Appendix*, Fig. S5*E*). In vitro droplet assays showed that all three deletion mutants retained strong phase-separation capacity, co-localized with G3BP1 in the presence of RNA, and enhanced G3BP1 droplet formation. In contrast, the KRmut completely failed to undergo phase separation, associate with G3BP1, or promote G3BP1 condensation (Fig. 5 *B* and *C* and *SI Appendix*, Fig. S5 *D* and *E*). Consistently, the FL Aurora-A KRmut also lost phase-separation ability and could not enhance G3BP1 droplet assembly (*SI Appendix*, Fig. S5 *H–J*). His-pull-down assays confirmed that, in the presence of RNA, the Δ2 to 48, Δ49 to 89, and Δ90 to 128 mutants bound G3BP1 at levels comparable to wild-type (WT) Aurora-A, whereas the KRmut showed no detectable interaction with the G3BP1–RNA complex (Fig. 5*D* and *SI Appendix*, Fig. S5*K*). NMR spectroscopy further demonstrated that the Aurora-A 1 to 128 KRmut failed to bind RNA, as no chemical-shift perturbations were observed upon RNA addition (Fig. 5*F* and *SI Appendix*, Fig. S5*G*), whereas the Δ2 to 48 mutant retained strong RNA-binding activity (Fig. 5*E* and *SI Appendix*, Fig. S5*F*).

In cellular contexts, Flag co-immunoprecipitation in HeLa cells revealed that Flag-tagged WT Aurora-A efficiently associated with G3BP1 upon sorafenib treatment, while the KRmut exhibited markedly reduced binding (Fig. 5*G*). Importantly, expression of the KRmut did not alter the levels of core SG components, sorafenib-induced eIF2α phosphorylation, or Aurora-A T288 phosphorylation (Fig. 5*I*), yet it significantly impaired Aurora-A phase separation and SGs assembly compared to WT Aurora-A (Fig. 5*H*). Consistent results were obtained in Hep-3B (*SI Appendix*, Fig. S5*L*) and PLC/PRF/5 (*SI Appendix*, Fig. S5*M*) cells, where the KRmut likewise failed to undergo phase separation and strongly suppressed SGs formation.

Collectively, these data show that deletion of individual IDR segments does not disrupt Aurora-A phase separation, whereas the positively charged K/R residues within the IDR are essential for RNA binding. These basic residues mediate the formation of an Aurora-A–RNA–G3BP1 ternary complex, which is required for efficient stress-granule assembly.

### Aurora-A-Orchestrated SGs Formation Drives Sorafenib Chemoresistance.

We next asked whether Aurora-A phase separation and its incorporation into SGs contribute to sorafenib resistance. To directly compare cells with and without Aurora-A condensation under identical conditions, we used a reduced sorafenib concentration (10 μM), at which approximately 40% of cells exhibited Aurora-A phase separation (*SI Appendix*, Fig. S1*A*). At this more physiological relevant concentration, which falls within the common experimental range, endogenous Aurora-A knockdown still suppressed SG formation (*SI Appendix*, Fig. S6*A*), and it was therefore used for subsequent survival assays.

**Fig. 6.**
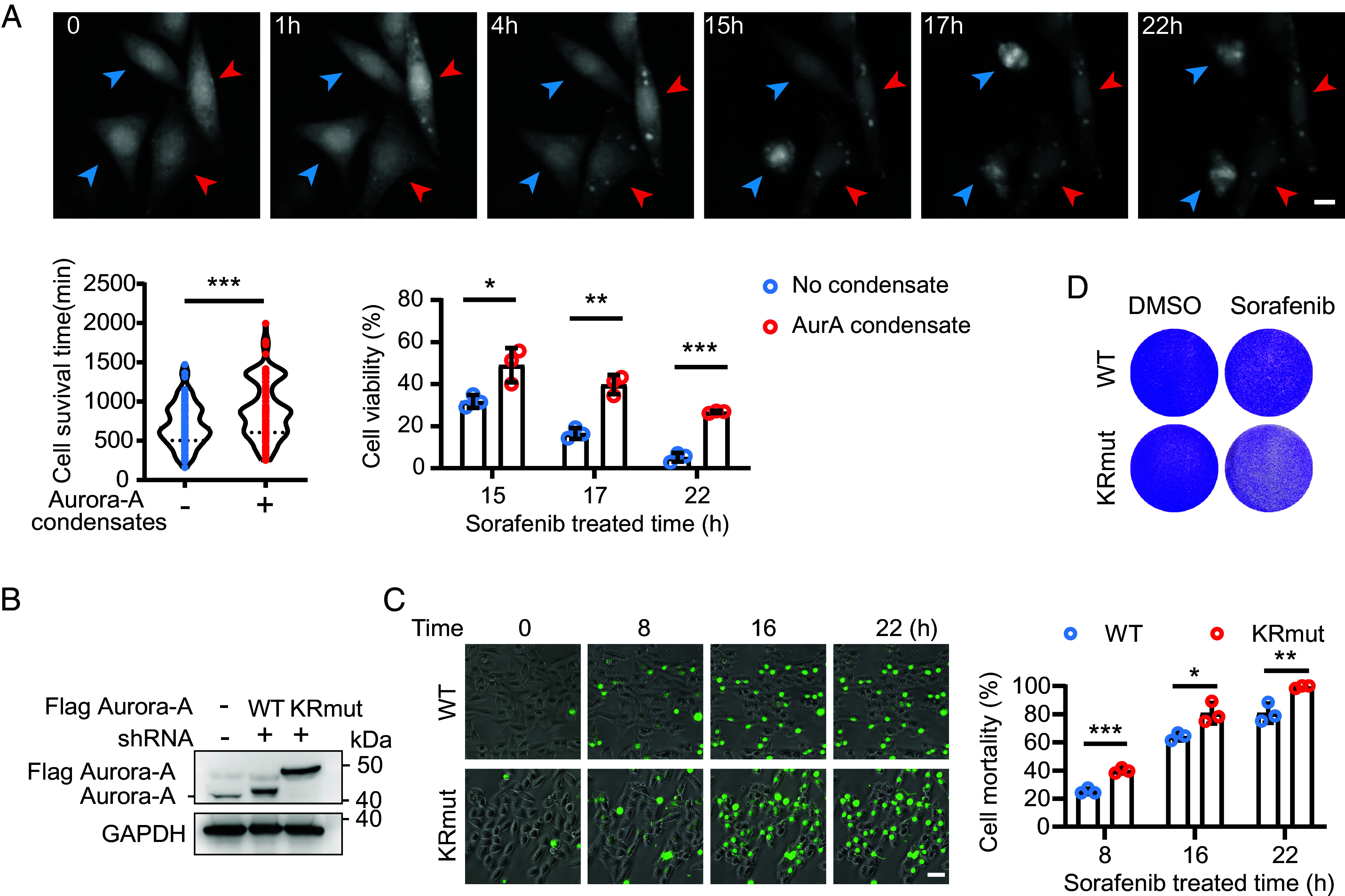
Aurora-A-orchestrated SGs formation drives sorafenib chemoresistance. (*A*) Time-lapse microscopy analysis compared the cell survival of HeLa cells with or without Aurora-A phase separation in the same field of view following 10 μM sorafenib treatment. Blue arrows indicate cells with no Aurora-A condensate; and red arrows indicate Aurora-A condensate cells. (Scale bar, 10 μm.) Quantification of the survival time of each cell (n = 100) and the cell survival rate at different time points following sorafenib treatment (n = 3). Mean ± SD, *P < 0.05; **P < 0.01; ***P < 0.001. (*B*) Validation of Flag-tagged FL Aurora-A wild type (Aurora-A-WT), Aurora-A KR amino acid mutant (Aurora-A-KRmut) overexpression and endogenous Aurora-A KD with shRNA in HeLa. (*C*) Time-lapse microscopy comparing HeLa cell survival between Aurora-A WT and KRmut treated with 10 μM sorafenib. SYTOX Green dye, labeling dead cells with green fluorescence. (Scale bar, 30 μm.) Quantification of cell mortality at 8 h, 16 h and 22 h post sorafenib treatment. n = 3, Mean ± SD, **P < 0.01; ***P < 0.001. (*D*) Crystal violet staining of HeLa Aurora-A WT or KRmut cell lines 2 d after treatment with DMSO or 10 μM sorafenib.

Live-cell imaging of HeLa cells expressing Aurora-A-mRuby2 showed that cells displaying Aurora-A phase separation survived significantly longer under sorafenib treatment than neighboring cells lacking condensates (Fig. 6*A*). Quantification of survival time and viability at different time points revealed a progressive decline in the survival of cells without Aurora-A condensates, whereas a substantial fraction of condensate-positive cells remained viable even at later time points (Fig. 6*A*). To establish causality, we knocked down endogenous Aurora-A in HeLa cells while re-expressing either WT Aurora-A or the phase-separation-deficient KR mutant. Under continuous sorafenib exposure, time-lapse imaging using SYTOX Green (a dye that labels dead cells) showed that KRmut-expressing cells died significantly more than WT-expressing cells (Fig. 6 *B* and *C*). Consistently, crystal violet staining demonstrated that cells expressing the Aurora-A KRmut were markedly more sensitive to sorafenib than those expressing WT Aurora-A, whereas basal cell growth was unaffected (Fig. 6*D*). Similar increases in sorafenib sensitivity were observed in hepatocellular carcinoma cell lines Hep-3B (*SI Appendix*, Fig. S6*B*) and PLC/PRF/5 (*SI Appendix*, Fig. S6*C*).

Together, these results indicate that Aurora-A phase separation—mediated by the basic K/R residues in its IDR—promotes SG assembly and enhances cellular tolerance to sorafenib, thereby driving chemoresistance.

### Aurora-A-Mediated SGs Formation Promotes Resistance to Sorafenib In Vivo.

**Fig. 7.**
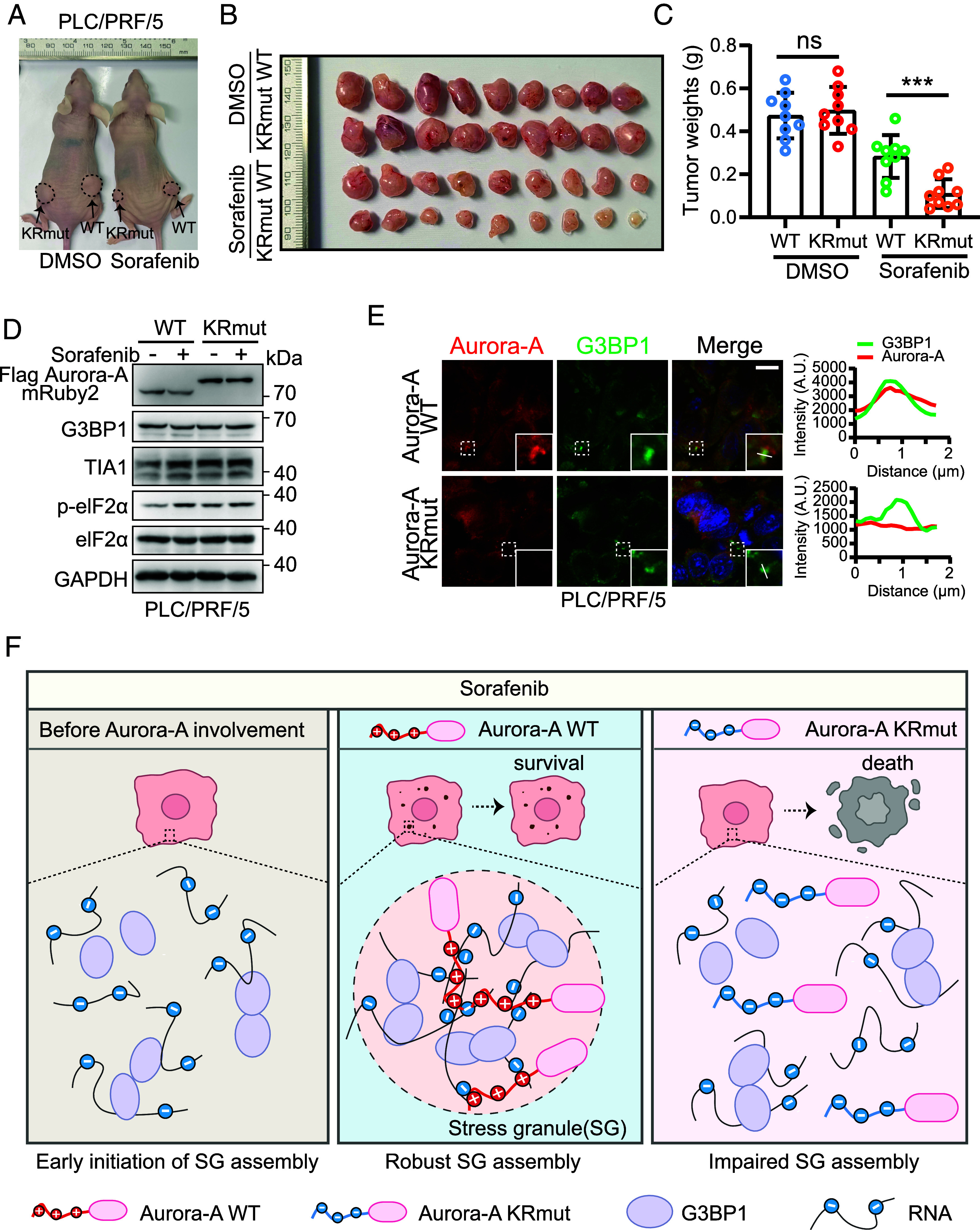
Aurora-A-mediated SGs formation promotes resistance to sorafenib in hepatocellular carcinoma. (*A*) Representative images of mice at 21 d post sorafenib administration. Sorafenib 30 mg/kg, i.g. (*B*) Photographs of PLC/PRF/5 xenograft tumors excised from individual mice. (*C*) The weight of excised PLC/PRF/5 xenograft tumors at 21 d post sorafenib administration. n = 9, Mean ± SD, ***P < 0.001; ns, nonsignificance. (*D*) Western blotting verified the expression of Aurora-A WT and KRmut in PLC/PRF/5 xenograft tumors derived from *B*. Sorafenib administration elevated eIF2α phosphorylation while leaving the total eIF2α protein level unchanged. (*E*) Immunofluorescence staining for G3BP1 and Aurora-A (WT or KRmut) in PLC/PRF/5 xenograft tumors derived from *B*. (*F*) Schematic drawing of sorafenib promotes cell formation of SGs, where wild type Aurora-A binds to RNA through the positively charged K/R residues in the N-terminal IDR, facilitating G3BP1 phase separation and SGs formation, promoting tumor cell resistance to sorafenib. However, Aurora-A KRmut cannot bind to RNA, making it difficult to form SGs necessary for resisting sorafenib stimulation.

To investigate the physiological relevance of this mechanism, we subcutaneously implanted nude mice with HeLa or PLC/PRF/5 cells expressing either WT Aurora-A or the phase-separation-deficient KR mutant. Mice were then treated with sorafenib or vehicle (Fig. 7*A* and *SI Appendix*, Fig. S7*A*). Sorafenib treatment partially suppressed tumor growth without affecting body weight (*SI Appendix*, Fig. S7*D*). Notably, under sorafenib treatment, tumors derived from KRmut-expressing cells were significantly smaller and lighter than those from WT controls, whereas no such differences were observed in vehicle-treated groups (Fig. 7 *B* and *C* and *SI Appendix*, Fig. S7 *B* and *C*). Immunoblot analysis of tumor lysates showed comparable levels of the SG components G3BP1 and TIA-1, as well as similar sorafenib-induced eIF2α phosphorylation, in WT and KRmut xenografts (Fig. 7*D* and *SI Appendix*, Fig. S7*F*). In contrast, immunofluorescence staining revealed a marked reduction in Aurora-A phase separation and its recruitment to SGs in KRmut tumors, which was accompanied by impaired SG assembly (Fig. 7*E* and *SI Appendix*, Fig. S7 *E*, *G*, and *H*).

Together, these in vivo data demonstrate that disrupting Aurora-A–mediated SGs formation through loss of phase separation sensitizes tumors to sorafenib, underscoring the pathophysiological importance of this mechanism in drug resistance.

## Discussion

Our study uncovers a kinase-independent role for Aurora-A in promoting SGs assembly and sorafenib resistance. We demonstrate that sorafenib treatment induces cytoplasmic phase separation of Aurora-A, which forms an RNA-dependent ternary complex with G3BP1 to nucleate and stabilize SGs, thereby enhancing tumor cell survival under drug stress (Fig. 7*F*). These findings establish Aurora-A as a critical scaffold for stress-adaptive cytoplasmic condensates.

Aurora-A is classically defined as a mitotic kinase whose functions are mediated through substrate phosphorylation (43). However, the limited clinical efficacy of catalytic Aurora-A inhibitors (44) underscores the importance of its non-catalytic roles. Prior studies have described kinase-independent functions of nuclear Aurora-A, such as regulating MYC transcription and alternative splicing (45, 46). Our work extends this paradigm by revealing that cytoplasmic Aurora-A binds RNA directly through its IDR, independent of kinase activity. By serving as an RNA-binding scaffold, Aurora-A promotes SGs formation through a direct interaction between its IDR and RNA (Fig. 7*F*); how this promotes further recruitment of G3BP1 remains to be defined.

The identification of Aurora-A as an RNA-binding protein expands its functional repertoire beyond canonical protein–protein interactions. Recent reports of RNA-mediated Aurora-A–substrate interactions during mitosis (47) support the broader relevance of RNA in regulating its functions. Although the precise RNA-recognition features remain to be defined, electrostatic complementarity between the positively charged IDR and the RNA phosphate backbone likely facilitates binding. Under basal conditions, Aurora-A appears to associate weakly with RNA; however, stress-induced phase separation, or its mitotic enrichment at spindle poles, may enhance RNA engagement (47). Whether and how stress or cell-cycle cues modulate this interaction warrants further investigation.

Consistent with an enhanced RNA-binding role under stress, sorafenib treatment promoted the co-fractionation of Aurora-A with translation-stalled mRNA (40S complexes) (*SI Appendix*, Fig. S2*F*). Aurora-A can undergo phase separation both at spindle poles during mitosis and in the cytoplasm under stress, yet the majority of Aurora-A within SGs is not phosphorylated (Fig. 3), indicating that condensation does not require kinase activity. It remains unclear whether Aurora-A phosphorylates substrates within SGs after nucleation.

Aurora-A-mediated SGs formation was observed under multiple stress conditions, including sorafenib treatment and heat shock, indicating a generalized stress-response mechanism. Despite distinct upstream stimuli, Aurora-A consistently localized to stress-induced condensates, suggesting convergence on common downstream phase separation pathways, potentially involving eIF2α signaling. Precisely how Aurora-A enhances G3BP1 phase separation merits further mechanistic study.

Our findings redefine Aurora-A as a multimodal regulator of SGs, whose kinase-independent, RNA-mediated phase separation activity complements its canonical mitotic roles. This mechanism provides insight into tumor adaptive resistance and may help explain the limited efficacy of catalytic Aurora-A inhibitors. Targeting IDR-mediated condensate formation, rather than kinase activity alone, may represent a promising therapeutic strategy to overcome sorafenib resistance while preserving essential mitotic functions.

## Materials and Methods

Detailed materials and methods are described in *SI Appendix*, *Materials and Methods*. Protocols are briefly described below.

### Cell Culture, Transfection, Cell Lysis, Immunoprecipitation and Immunoblotting, Immunofluorescence Imaging.

Cell culture, transfection, cell lysis, immunoprecipitation and immunoblotting, immunostaining, and confocal microscopy were performed as previously published (48). Cell lines and tumor tissue images were acquired with DeltaVision softWoRx software (version 6.5.2) and processed by deconvolution and z-stack projection. Detailed information for antibodies used is listed in *SI Appendix*, Table S2.

### Quantification of SGs.

Cell with SGs was calculated as the ratio of SG-positive cells to the total cell number (22). SGs area per cell was quantified as the ratio of SG area to total cell area in individual cells (49).

### Cloning, Protein Expression, and Purification.

Detailed information on strains and plasmids used in this study is listed in *SI Appendix*, Table S3. The purification buffer consisted of 2 M NaCl, pH 7, and 20 mM Na_2_HPO_4_. Purification was carried out using Ni-NTA affinity chromatography. Detailed information in *SI Appendix*.

### Droplets and In Vitro Kinase Assays.

Alexa Fluor™ 488 and 594 fluorescent dyes are used to label hG3BP1 and hAurora-A (or various truncated and mutant forms of hAurora-A), respectively. Various proteins are prepared using a phosphate-buffer (150 mM NaCl, pH 7, 20 mM Na_2_HPO_4_). Imaging is performed using a Zeiss LSM 980 laser scanning confocal microscope with 100× oil objective. The partition coefficient (K) was calculated as described (39, 50).

### His Pull Down.

The His-tagged proteins hAurora-A or various mutations were incubated with cOmplete His-Tag purification resin and washed in phosphate buffer (20 mM Na_2_HPO_4_ 150 mM NaCl, 3 mM imidazole, 1% NP-40, pH 7.0). 200 μg of hG3BP1 and 200 μg of total RNA were added.

### NMR Spectroscopy.

The total sample volume was 500 μL (including 10% D_2_O), with the concentration of ^15^N-labeled hAurora-A-IDR and its various mutations being 100 μM. ^1^H-^15^N HSQC spectra were acquired at 25 °C on an Agilent 700 MHz spectrometer (Agilent Technologies, Santa Clara, CA).

### Live-Cell Imaging of Phase Separation.

HeLa (Aurora-A mRuby2) stable cell lines were seeded onto an eight-chambered coverglass (Ibidi, 80826). Images were acquired every 5 to 10 min with 488 nm and 594 nm lasers using the Nikon Eclipse Ti-E at 37 °C.

### Cell Viability Analysis.

Cell survival time was quantified as the duration of individual cell survival. Cell viability was determined by the ratio of viable cells to the total cell number at different time points.

### SYTOX Green Analysis.

0.1 μM SYTOX Green was added. Images were acquired using the Nikon Eclipse Ti-Eat 37 °C. Cell mortality was quantified as the ratio of green-positive cells to the total number of cells (51).

### Mouse Xenograft Experiments.

For the mouse xenograft experiments, 5 × 10^6^ HeLa or 1 × 10^7^ PLC/PRF/5 cells were injected subcutaneously into the left or right flank of each mouse (33). 30 mg/kg sorafenib is administered orally once a day.

### Quantification and Statistical Analysis.

The definitions and exact values of n, distributions, and deviations form experiments are presented in the corresponding Figure Legends. Unless stated otherwise within the text, statistical analysis was performed using GraphPad Prism. Error bars for all data represent SD. Statistical significance was assessed by two-way ANOVA and unpaired two-tailed Student’s *t* test. Statistical significance is displayed as ns, no significance; *P < 0.05; **P < 0.01 and ***P < 0.001.

## Supplementary Material

Appendix 01 (PDF)

## Data Availability

Study data are included in the article and/or *SI Appendix*.
