# Supplementary material for: Aurora-A drives sorafenib resistance by scaffolding stress granule assembly via phase separation
Source: Proc Natl Acad Sci U S A. 2026 Apr 20;123(17):e2516469123. doi: 10.1073/pnas.2516469123 (PMC13123825; doi:10.1073/pnas.2516469123)
Supplement: Supplementary file 1 — Appendix 01 (PDF) [file pnas.2516469123.sapp.pdf]

Supporting Information for

Aurora-A Drives Sorafenib Resistance by Scaffolding Stress Granule Assembly via Phase Separation

Lingyu Kong[1,#], Fumei Zhong[2,3,#], Fazhi Yu[1,#], Ting Wang[1], Gang Wang[1], Yu Bai[1], Han Xia[1], Zihang Pan[1], Mingxue Liu[1], Yan Zhang[1], Rui Feng[1], Jiahai Zhang[2], Yingying Du[4], Kaiguang Zhang[1,*], Jing Guo[1,*], Ke Ruan[2,5,*], Zhenye Yang[1, 2,5,6,*]

[1]Department of Digestive disease, The First Affiliated Hospital of USTC, State Key Laboratory of Immune Response and Immunotherapy, Division of Life Sciences and Medicine, University of Science and Technology of China, Hefei, 230027, China.

[2]MOE Key Laboratory for Cellular Dynamics and Membraneless Organelle, Division of Life Sciences and Medicine, University of Science and Technology of China, Hefei, 230027, China.

[3]GMU-GIBH Joint School of Life Sciences, Guangdong Provincial Key Laboratory of Protein Modification and Disease, The Guangdong-Hong Kong-Macao Joint Laboratory for Cell Fate Regulation and Diseases, Guangzhou Medical University，Guangzhou，511436，China.

[4]Department of Oncology, the First Affiliated Hospital of Anhui Medical University, Hefei, 230022, China.

[5]Center for Advanced Interdisciplinary Science and Biomedicine of IHM, Division of Life Sciences and Medicine, University of Science and Technology of China.

[6]Institute of Cancer Research, Anhui Key Laboratory of Molecular Oncology, Division of Life Sciences and Medicine, University of Science and Technology of China, Hefei 230026, China

# These authors contributed equally to this work.

Correspondence: University of Science and Technology of China, 443 Huangshan Rd, Hefei, Anhui, China 230027; Phone: 86-551-63600613; Fax: 86-551-63601443.

Email: Zhenye Yang: zhenye@ustc.edu.cn; Ke Ruan: kruan@ustc.edu.cn; Jing Guo: jguo2013@ustc.edu.cn or Kaiguang Zhang zhanghaiguang@ustc.edu.cn

This PDF file includes:

Supporting text (Supplementary Materials and Methods)
Figures S1 to S7
Tables S1 to S3
SI References

Materials and Methods

Cell Culture, Transfection

PLC/PRF/5 cells were a gift from Prof. Huafeng Zhang at the University of Science and Technology of China. Detailed information for cell lines and reagents used is listed in Table S1. Cells were cultured in Dulbecco modified Eagle media (Gibco) supplemented with 10% FBS. Lentivirus shRNA production, viral infection, and stable knockdown, overexpression cell selections were performed using psPAX2 and pMD2.G in a 2:1:1 ratio(1).

Construction of knockout cell lines

For constructing CRISPR-Cas9-mediated G3BP1/2 double knockout (DKO) cells, lentiCas9-Blast (Addgene, 52962) was transfected into HEK293T cells along with psPAX2 and pMD2.G in a 2:1:1 ratio. The cells were treated with 10 μg/ml blasticidin. Blasticidin-resistant cells were subsequently transduced with lentivirus carrying guide RNA sequences targeting both G3BP1 and G3BP2(2). Stably transfected cells were selected using 1 μg/ml puromycin, and different G3BP1/2 DKO clones were analyzed by immunoblotting.

Cell lysis, immunoprecipitation and immunoblotting

The cells were lysed using a buffer composed of 50 mM Tris-HCl (pH 7.4), 150 mM NaCl, 50 mM NaF, 1 mM EDTA, 1 mM $Na_2P_2O_4$, 1 mM Na_3VO_4, 1 mM PMSF, and a protease inhibitor cocktail (Sigma). To perform immunoblotting, the samples, which were either immunoprecipitates or whole-cell lysates, were separated on 10-12% SDS-polyacrylamide gels after being boiled at 95 °C for 5 minutes. The proteins were then transferred onto 0.22-μm PVDF membranes (Millipore). After blocking with 5% non-fat milk in Tris-buffered saline with 0.1% Tween-20 for 30 minutes to 1 hour, the membranes were incubated with primary antibodies at 4 °C overnight and with secondary antibodies for 1 hour at room temperature. Detection of signals was achieved using Western Lightning Chemiluminescence Reagent Plus, and ChemiScope software (version 17.05.17A) was used for analysis. ImageJ was used to perform the quantitative analysis. Detailed information for antibodies used is listed in Table S2.

Immunofluorescence

The cells were fixed with 4% paraformaldehyde (PFA) for 15 min at RT. Next, the cells were incubated with 0.2% Triton X-100 in PHEM for 5 min and then blocked with 1% BSA in TBST for 0.5-1 h. Primary antibodies were incubated for 2 h at RT followed by secondary antibody incubation and DAPI staining. Finally, the cells were mounted with Dako Fluorescence Mounting Medium (Dako North America, S302380-2). Images were acquired with DeltaVision softWoRx software (version 6.5.2) and processed by deconvolution and z-stack projection.

Plasmids

For RNA interference-based knockdown experiments, The Aurora-A targeting sequence (shRNA1:5'-GCAAGCAGCCCCTGCCATCGG-3' and shRNA2:5'-CCTGTCTTACTGTCATTCGAA-3') was inserted into pLKO.1 (Addgene) or pTripZ Inducible Lentiviral vector, which is inducible with doxycycline. Flag-Aurora-A mRuby2 was constructed by cloning the sequence for full-length Aurora-A into the XbaI and BamHI sites of pLVX

(Clontech). Aurora-A D274A, KRmut and truncated mutants were constructed by cloning sequences for Flag-Aurora-A D274A, Flag-Aurora-A KRmut and Aurora-A with deletion of amino acids 2-48, 49-89, 90-128 into the XbaI and BamHI sites of pLVX, or into pET-22b (Addgene). Oligonucleotide sequence of shRNAs, sgRNAs, and primers for PCR used is listed in Table S3.

Cloning, Protein Expression, and Purification

The following constructs: hAurora-A, hAurora-A Δ2-48, hAurora-A Δ49-89, hAurora-A Δ90-128, hAurora-A IDR (1-128), hAurora-A IDR Δ2-48, hAurora-A IDR Δ49-89, hAurora-A IDR Δ90-128, various mutants of hAurora-A IDR, hAurora-A 129-403, hG3BP1, and hG3BP1 RRM-RGG, were cloned into the pET22b vector and transformed into Escherichia coli BL21(DE3) competent cells for recombinant protein expression. When the optical density of the bacterial culture at 600 nm (OD600) reached 1.0-1.2, protein expression was induced by adding 0.3 mM isopropyl β-D-1-thiogalactopyranoside (IPTG), followed by shaking culture at 16°C for 24 hours. For ^{15}N isotopic labeling, hAurora-A IDR and its mutants were expressed in LR medium supplemented with 0.5 g/L $^{15}NH_4Cl$ as the sole nitrogen source. All proteins were purified using a buffer consisting of 20 mM Na_2HPO_4 (pH 7.0) and 2 M sodium chloride. Initial purification was performed via Ni^{2+}-nitrilotriacetic acid (Ni-NTA) affinity chromatography. Purification was carried out using Ni-NTA affinity chromatography. Depending on the needs of subsequent experiments, the His tag was either cleaved by TEV protease or retained. and the proteins were further purified by size-exclusion HiLoad16/600 Superdex75/200 column. Finally, the proteins were dialyzed into a buffer containing 150 mM NaCl, pH 7, and 20 mM Na_2HPO_4, concentrated, and stored at -80 °C until use.

RNA extraction

Total RNA is extracted from human-derived HeLa cells using the SteadyPure Universal RNA Extraction Kit (AG, AG21017). The RNA is then dissolved in DEPC water and the concentration is measured using a One Drop device. The RNA is stored at -80 ℃ until further use.

Droplets and in vitro kinase assays

Proteins were fluorescently labeled for visualization purposes: hG3BP1 was labeled with Alexa Fluor™ 488, and hAurora-A (including its various truncated and mutant forms) was labeled with Alexa Fluor™ 594. After the labeling reaction, unbound dye molecules were removed via dialysis. All proteins were diluted to their specified concentrations in phosphate-buffered saline (20 mM Na_2HPO_4, 150 mM NaCl, pH 7.0). Similarly, RNA stock solutions were prepared in the same buffer. For the phase separation assay, proteins and 50 ng/μL total RNA were mixed at the desired concentrations in a final volume of 10 μL(2). To examine the effect of kinase activity on condensates, 200 μM ATP and 200 μM magnesium chloride (pre-diluted from stock solutions in the aforementioned phosphate buffer) were included in the reaction mixture. Additionally, 1 μM Aurora-A or 100 μM sorafenib (pre-diluted from a stock solution in the same phosphate buffer) was either added or excluded according to experimental requirements. Imaging was conducted using a Zeiss LSM 980 laser scanning confocal microscope equipped with a 100× oil immersion objective.

The partition coefficient (K) was calculated using the formula: K= Aurora-A droplets (the mean

fluorescence intensity of Aurora-A inside the droplets)/Aurora-A bulk (the mean fluorescence intensity of Aurora-A in the background bulk aqueous phase outside the droplets)(3, 4). Droplets area were quantified based on the area of GFP^+ G3BP1 droplets.

His pull down

The proteins hAurora-A or various truncated or mutated forms of hAurora-A, each carrying a His tag (200 μg), were incubated with cOmplete His-Tag purification resin at 4 °C for 2 hours. Subsequently, the resin was washed three times with a phosphate buffer (20 mM Na_2HPO_4, 150 mM NaCl, 3 mM imidazole, 1% NP-40, pH 7.0) to remove unbound proteins. Then, 200 μg of hG3BP1 and 200 μg of total RNA were added, along with a control group that did not receive total RNA. After overnight incubation at 4 °C, the resin was washed again three times with the phosphate buffer. Samples were taken for SDS-PAGE electrophoresis analysis.

NMR spectroscopy

The total sample volume was 500 μL (including 10% D_2O), with the concentration of ^{15}N-labeled hAurora-A-IDR and its various mutant or truncated proteins being 100 μM. ^{1}H-^{15}N HSQC spectra were acquired at 25 °C on an Agilent 700 MHz spectrometer (Agilent Technologies, Santa Clara, CA). Data were collected with 64 scans and 128 complex points per increment, using a 1.3 s relaxation delay, resulting in a total acquisition time of 1.5 hours per spectrum(5). All two-dimensional spectra were processed using NMRPipe 1 and visualized with Sparky 2.

Polysome profiling

HeLa WT cells (2×10^7 cells per group) were treated with DMSO, sorafenib (200 μM for 2 h), or sorafenib (200 μM for 2 h) plus cycloheximide (CHX, 100 μg/mL for 2 h). After treatment, cells were harvested by trypsinization and centrifugation at 1,200 × g for 5 min, washed once, and resuspended in 1× PBS. Cell suspensions were snap-frozen in liquid nitrogen and submitted to Bluescape Hebei Biotech Co., Ltd. (Baoding, China) for polysome profiling analysis. A portion of total cell lysate was used as input. Fractions collected from each sample were quantified by Bradford assay and analyzed by SDS–PAGE(6, 7).

Crystal violet staining

Wash the cells with PBS and fix them with 4% PFA for about 15 mins at RT. Remove the fixative and add crystal violet stain for 30 mins. Wash until the wash solution is clear then allow the plates to air dry completely.

SYTOX Green analysis

HeLa (Aurora-A WT/KRmut) stable cell lines were seeded onto an eight-chambered coverglass (Ibidi, 80826). 0.1 μM SYTOX Green was added. Images were acquired every 10 min with 488nm laser after 10μM sorafenib treatment using the Nikon Eclipse Ti-E with NIS-Elements AR software (version 4.30.1.10210) at 37 °C. The image sequences were viewed and analyzed using ImageJ. Cell mortality was quantified as the ratio of green-positive cells to the total number of cells(8).

Mouse xenograft experiments

Six-week-old BALB/c nude mice (randomized by sex.) were purchased from Shanghai SLAC Laboratory Animal. For the mouse xenograft experiments, 5×10^6 HeLa or 1×10^7 PLC/PRF/5 Flag Aurora-A WT or KRmut cells were injected subcutaneously into the left or right flank of each mouse(9). 30 mg/kg sorafenib is administered orally once a day. The xenograft tumour burden was less than the maximum tumour size (1 cm^3) approved by the Animal Research Ethics Committee of the University of Science and Technology of China (USTCACUC27110122054).

Sequence analysis

The PONDR program (http://www.pondr.com/) was used to analyze disordered regions of Aurora-A. Two indicators (VLS2 and VL3) showed Aurora-A possesses intrinsic disordered region. The homologous sequences of Aurora-A, Aurora-B, and Aurora-C were analyzed using the MultAlin tool, available at http://multalin.toulouse.inra.fr/multalin. The net charge per residue of Aurora-A full long and IDR sequence were analyzed using CIDER, available at https://pappulab.wustl.edu/CIDER/.

Quantification and statistical analysis

The definitions and exact values of n, distributions and deviations form experiments are presented in the corresponding Fig. Legends. Unless stated otherwise within the text, statistical analysis was performed using GraphPad Prism. Error bars for all data represent SD. Statistical significance was assessed by two-way ANOVA for Figs. S2B, S2D, S2E, 3E, S4B, S5E, S5J, 6A right, 6C; by unpaired two-tailed Student's t-test for Figs. 1A, 1B, S1D, 2B, 2C, 3C, S3C, 4B, S4A, S4K, S4N, 5H, S5D, S5I, S5L, S5M, 6A left, 6E, S6A, 7C, S7C, S7D. Statistical significance is displayed as ns, no significance; $^{*}P < 0.05$; $^{**}P < 0.01$ and $^{***}P < 0.001$.

Supplemental Figures

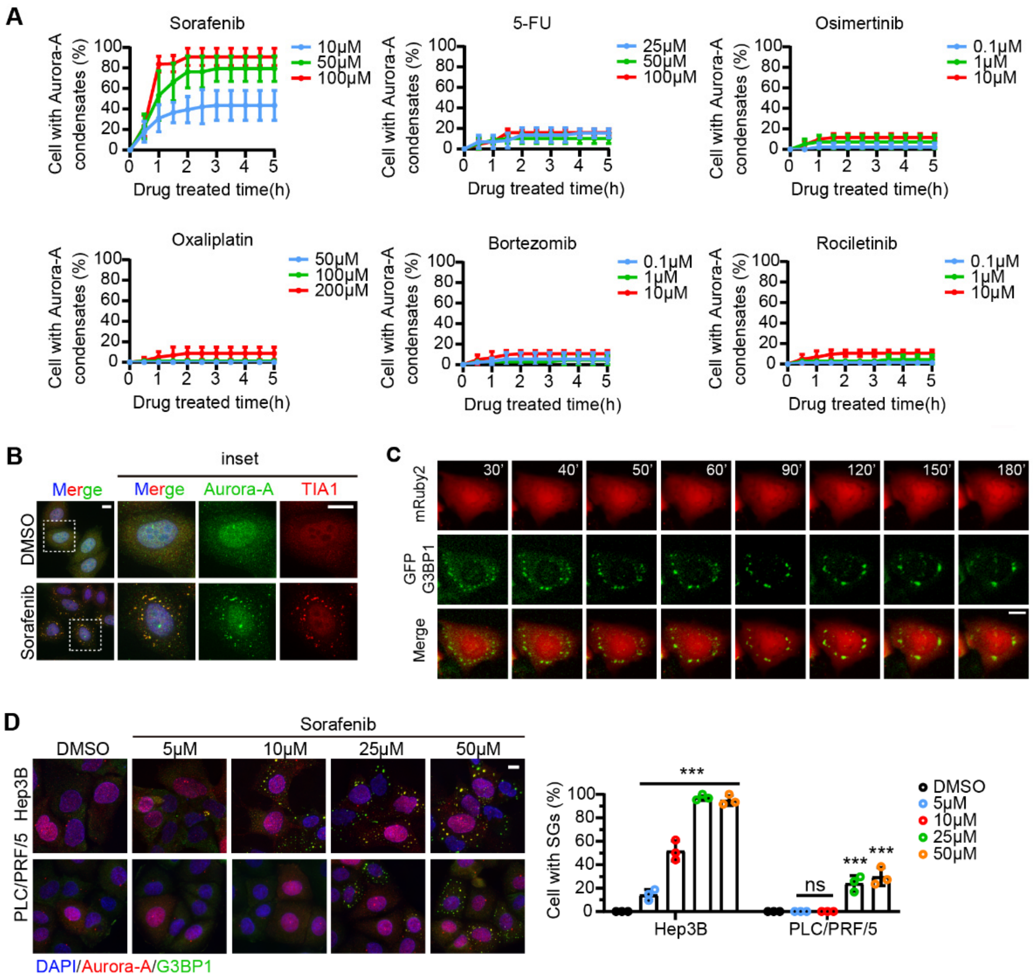

Figure S1. Sorafenib triggers Aurora-A phase separation and co-localization with SGs.

A. Aurora-A mRuby2 was visualized via time-lapse microscopy in HeLa. Quantification of the proportion of cells with Aurora-A granule formation at different time points following treatment with three concentrations of the drugs, respectively. n= 3 biologically independent experiments.

B. Protein localization assessed by immunofluorescence analysis for Aurora-A (green) and TIA1(red) in HeLa cells that were treated with dimethyl sulfoxide (DMSO), 100 μM sorafenib (2 h). Nucleus was stained with DAPI (blue). Scale bar, 10 μm. Magnification of the demarcated region was shown as the inset from the merged images. Inset scale bar, 10 μm.

C. Time-lapse microscopy of mRuby2 and GFP-G3BP1 granule dynamics with 100 μM sorafenib treated. Scale bar, 10 μm.

D. Confocal imaging of Hep-3B and PLC/PRF/5 hepatocellular carcinoma cells treated with sorafenib at different concentrations for 2 h was performed to visualize SGs formation. Quantification of the proportion of cells with SGs formation under different concentrations of sorafenib treatment (n=3). Mean ± SD, ***p<0.001; ns, non-significance.

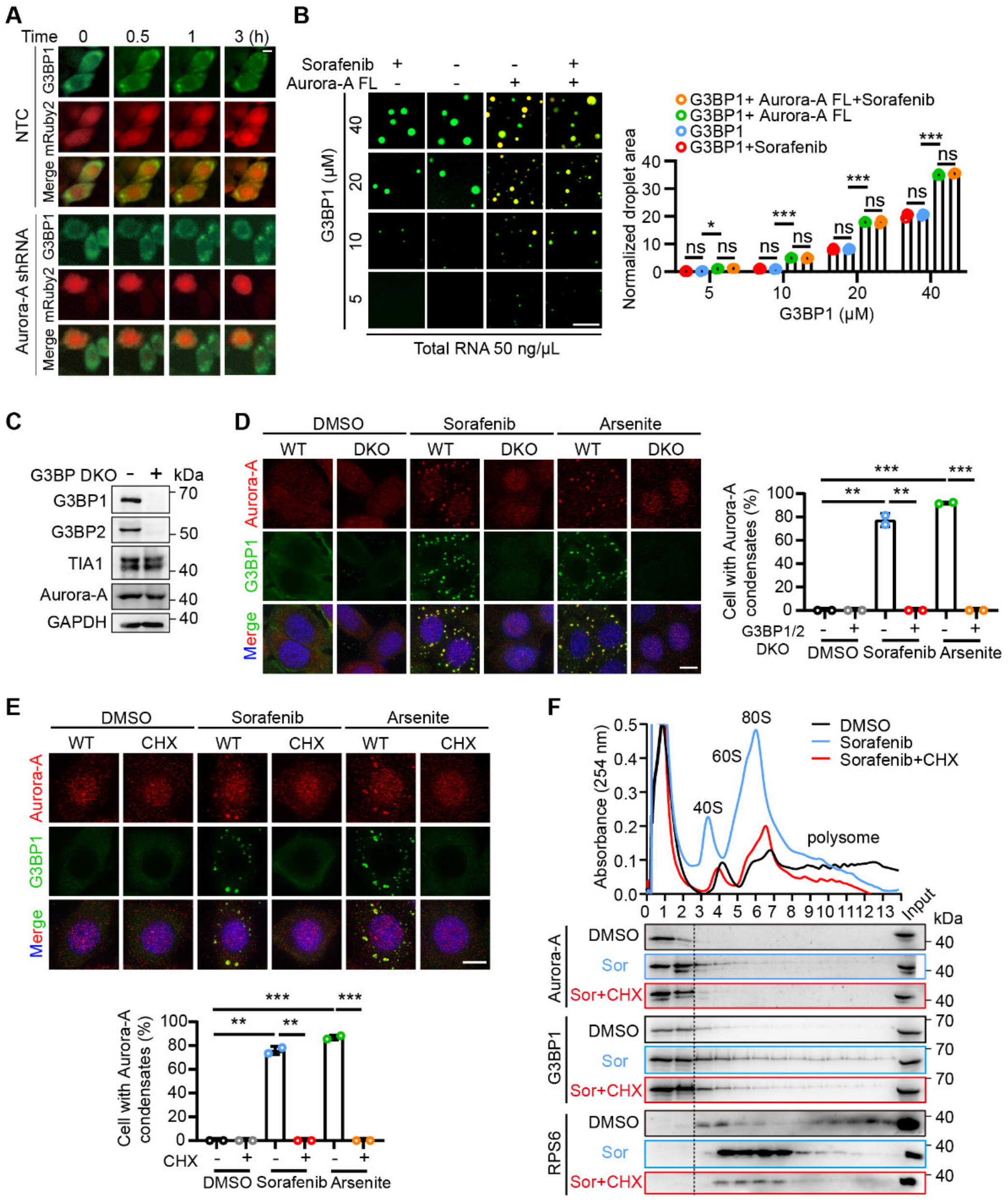

Figure S2. Aurora-A phase separation is dependent on SGs formation.

A. Time-lapse microscopy revealed that cells with doxycycline-induced Aurora-A shRNA, which exhibited red fluorescence, showed a significant reduction in SGs formation (GFP-G3BP1, green) under 100 μM sorafenib treatment. Scale bar, 10 μm.

B. The addition of sorafenib in vitro does not affect the formation of Aurora-A-G3BP1 droplets. Confocal microscopy images show the coexistence of Alexa Fluor™ 488-labeled G3BP1 (green) and Alexa Fluor™ 594-labeled Aurora-A (red). The experiment was performed using purified proteins in a buffer containing of 20 mM Na_2HPO_4, 150 mM NaCl, (pH 7.0), 50 ng/μL total RNA, 200 μM ATP, and 200 μM $MgCl_2$, with the addition of 1 μM Aurora-A, or 100 μM sorafenib was either included or excluded as needed. Images show

representative data from three independent experiments. Scale bar, 10 μm. Quantification of the area of G3BP1 droplets. Mean ± SD, *p<0.05; ***p<0.001; ns, non-significance.

C. Western blotting showing the level changes of endogenous G3BP1, G3BP2 in HeLa cells upon G3BP knock out with sgRNA. Knockout of G3BP has no effect on the expression level of Aurora-A and other SGs proteins TIA1.
D. Immunofluorescence analysis of Aurora-A phase separation under sorafenib or arsenite treatment in G3BP1/2 DKO cells. Scale bar, 10 μm. Quantification of the proportion of cells with Aurora-A granule formation. n=2, Mean ± SD, **p<0.01; ***p<0.001.
E. Immunofluorescence analysis for Aurora-A phase separation combined with cycloheximide (CHX) and sorafenib or arsenite. Scale bar, 10 μm. Quantification of the proportion of cells with Aurora-A granule formation. n=2, Mean ± SD, **p<0.01; ***p<0.001.
F. Polysome profiling of HeLa cells treated with DMSO, 100 μM sorafenib 2h, or 100 μM sorafenib plus 100 μg/mL CHX 2h. Western blotting showing the level changes of G3BP1 and Aurora-A. RPS6, small ribosomal subunit member.

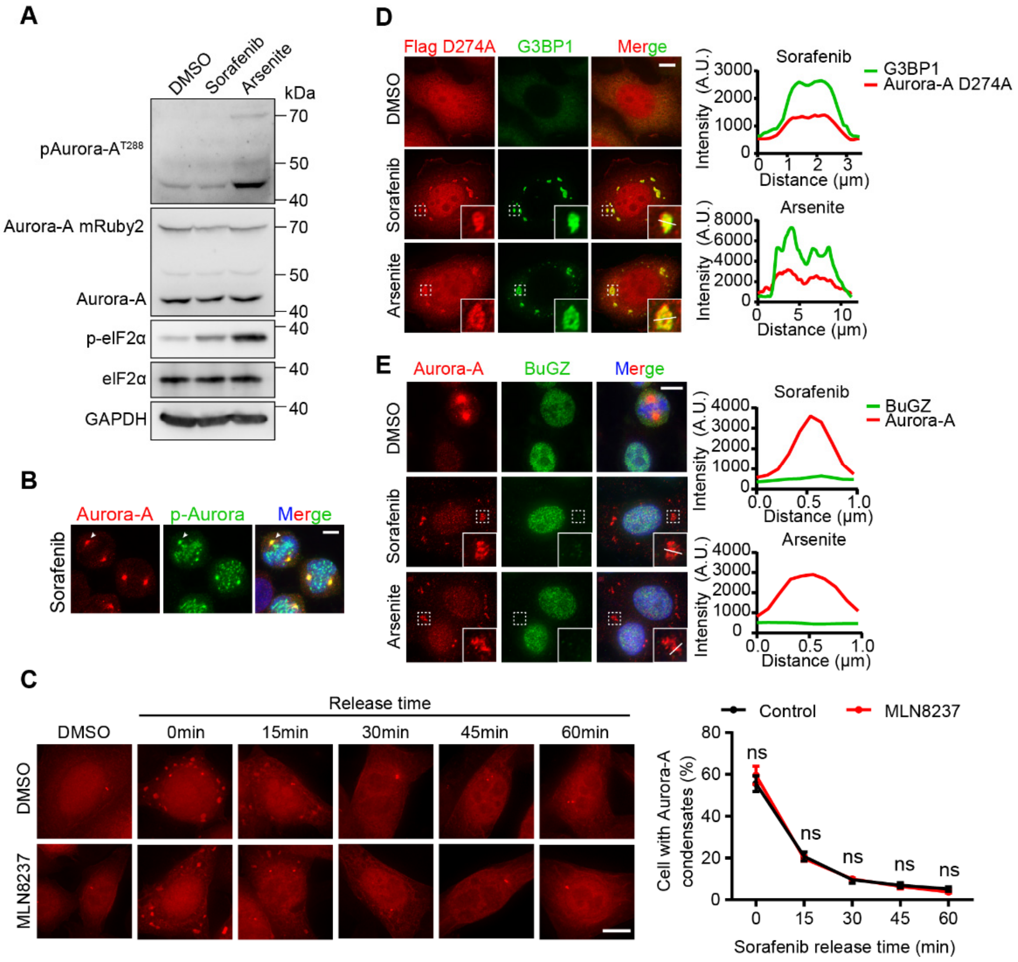

Figure S3. Aurora-A promotes SGs formation independently of its kinase activity and BuGZ.

A. Western blotting showing the level changes of endogenous Aurora-A and exogenous Aurora-A-mRuby2 in HeLa cells that were treated with DMSO, 100 μM sorafenib (2 h), 100 μM arsenite (1 h)

B. Protein localization assessed by immunofluorescence analysis for Aurora-A and p-Aurora in HeLa cells that were treated with 100 μM sorafenib (2 h). Arrows indicate the normal centrosomal accumulation of p-Aurora. Scale bar, 10 μm.

C. Time-lapse microscopy of Aurora-A-mRuby2 granule dynamics with 100 μM sorafenib and 100 nM MLN8237 treated. Scale bar, 10 μm. Quantification of the proportion of cells with Aurora-A granule formation under different times of sorafenib treatment. n=3, Mean ± SD, ns, non-significance.

D. Protein localization assessed by immunofluorescence analysis for Flag-Aurora-A D274A(Flag) and G3BP1 in HeLa cells that were treated with DMSO, 100 μM sorafenib (2 h), 100 μM arsenite (1 h). Scale bar, 10 μm. Profile intensity showing Flag-Aurora-A D274A /G3BP1 fluorescence signals of the white boxs for each condition from the merged images.

E. Protein localization assessed by immunofluorescence analysis for Aurora-A and BuGZ in HeLa cells that were treated with DMSO, 100 μM sorafenib (2 h), 100 μM arsenite (1 h).

Nucleus was stained with DAPI (blue). Scale bar, 10 μm. Profile intensity showing Aurora-A/BuGZ fluorescence signals of the white boxs for each condition from the merged images.

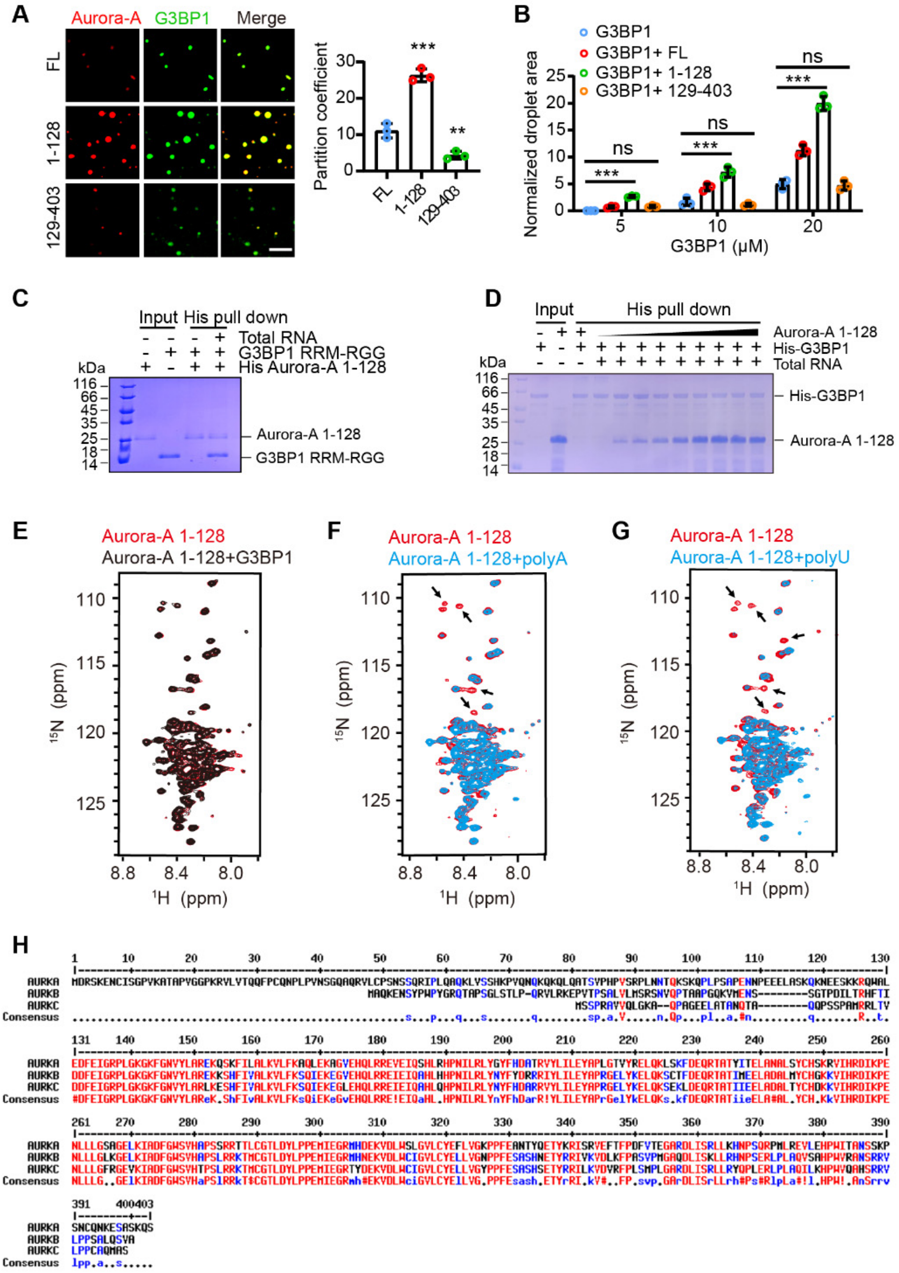
A
Aurora-A
G3BP1
Merge
FL
1-128
129-403
Partition coefficient
***
**
B
G3BP1
G3BP1+ FL
G3BP1+ 1-128
G3BP1+ 129-403
Normalized droplet area
ns
***
G3BP1 (μM)
C
Input
His pull down
Total RNA
G3BP1 RRM-RGG
His Aurora-A 1-128
kDa
Aurora-A 1-128
G3BP1 RRM-RGG
D
Input
His pull down
Aurora-A 1-128
His-G3BP1
Total RNA
kDa
His-G3BP1
Aurora-A 1-128
E
Aurora-A 1-128
Aurora-A 1-128+G3BP1
F
Aurora-A 1-128
Aurora-A 1-128+polyA
G
Aurora-A 1-128
Aurora-A 1-128+polyU
15N (ppm)
1H (ppm)
H
AURKA
AURKB
AURKC
Consensus

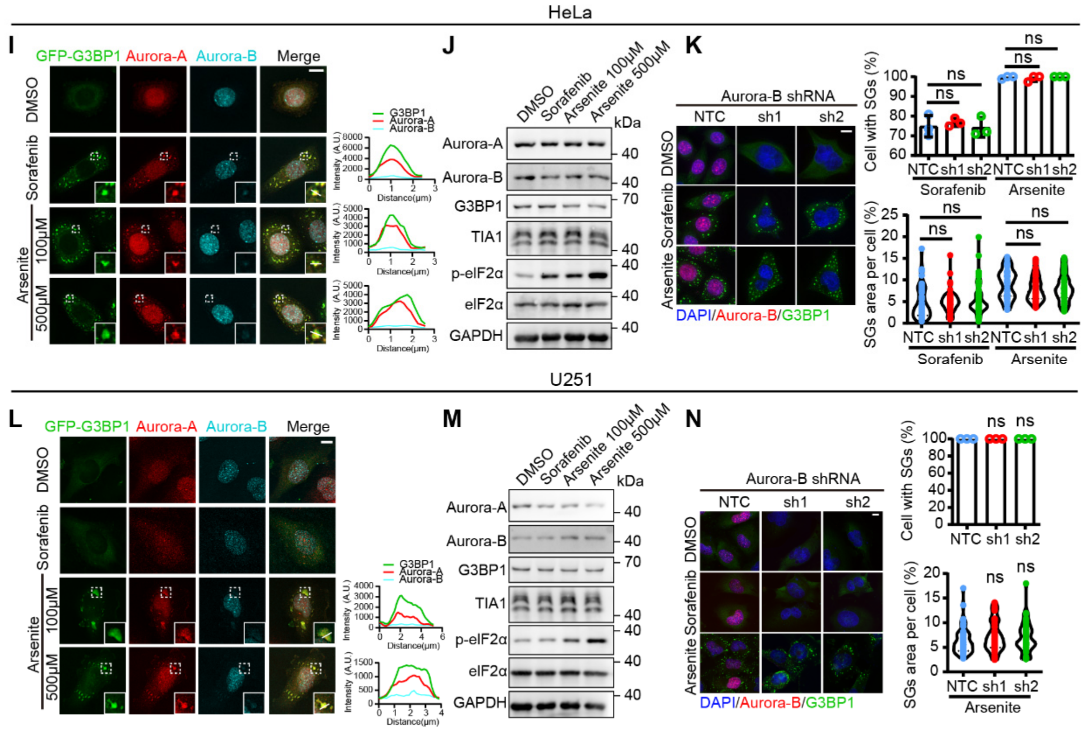

Figure S4. Aurora-A IDR interacts directly with RNA instead of G3BP1.

A. Phase separation of 1 μM Alexa Fluor™ 594-labeled Aurora-A full-length (FL), 1-128 or 129-403 (red) with 20 μM Alexa Fluor™ 488-labeled G3BP1 (green) in the presence of 50 ng/μL total RNA. The assay was performed using purified recombinant proteins in phosphate buffer (20 mM Na_2HPO_4, 150 mM NaCl, pH 7.0). Images show representative data from three independent experiments. Scale bar, 10 μm. Quantification of the Aurora-A FL, 1-128 or 129-403 partition coefficient from three independent measurements. Mean ± SD, **p<0.01; ***p<0.001.

B. Quantification of the area of G3BP1 droplets in Fig. 4C. n=3, Mean ± SD, ***p<0.001; ns, non-significance.

C. His pull-down assay demonstrated an RNA-dependent interaction between Aurora-A 1-128 and the G3BP1 RRM-RGG domain. Purified His-tagged Aurora-A 1-128 (200 μg) was incubated with untagged G3BP1 RRM-RGG domain (200 μg) in the presence or absence of total RNA (200 μg). Proteins bound to Ni-NTA beads were analyzed by Coomassie blue staining.

D. His pull-down of purified 200 μg His-tagged G3BP1 in complex with 0, 0.5, 1, 2, 4, 8, 16, 32, and 64-fold excess of Aurora-A 1-128 in the presence of 200 μg total RNA, the pull-down of Aurora-A 1-128 increases as the concentration rises and eventually reaches saturation.

E. Overlay of ^{1}H-^{15}N-HSQCs panorama of either 100 μM Aurora-A 1-128 alone (red) or in complex with a 1:1 ratio of G3BP1 (black).

F. Overlay of ^{1}H-^{15}N-HSQCs panorama of either 100 μM Aurora-A 1-128 alone (red) or in complex with a 1:1 ratio of 25nt-polyA (blue). Selected residues experiencing large chemical shift perturbations are labeled with arrows.

G. Overlay of ^{1}H-^{15}N-HSQCs panorama of either 100 μM Aurora-A 1-128 alone (red) or in

complex with a 1:1 ratio of 15nt-polyU (blue). Selected residues experiencing large chemical shift perturbations are labeled with arrows.

H. Conservation of homologous sequences between Aurora-A, Aurora-B and Aurora-C, aligned using MultAlin.

I. Protein localization assessed by immunofluorescence analysis for Aurora-A, Aurora-B and G3BP1 in HeLa cells that were treated with DMSO, 100 μM sorafenib (2 h), 100 μM arsenite (1 h), 500 μM arsenite (30 min). Scale bar, 10 μm. Profile intensity showing Aurora-A/Aurora-B/G3BP1 fluorescence signals of the white lines for each condition from the merged images.

J. Western blotting showed that the protein expression levels of Aurora-A, Aurora-B, and the SGs core proteins G3BP1, TIA1 unchanged, whereas the phosphorylation level of eIF2α was increased in HeLa cells with 100 μM sorafenib (2 h), 100 μM arsenite (1 h), 500 μM arsenite (30 min).

K. Confocal imaging of non-target control (NTC) and Aurora-B shRNA1, shRNA2 treated with 100 μM sorafenib (2 h) , and 500 μM arsenite (30 min) to visualize the formation of SGs in HeLa cells. Quantification of the proportion of cells with SGs formation (n=3) and the area of SGs per cell (n=50). Mean ± SD, ns, non-significance.

L. Protein localization assessed by immunofluorescence analysis for Aurora-A, Aurora-B and G3BP1 in U251 cells that were treated with DMSO, 100 μM sorafenib (2 h), 100 μM arsenite (1 h), 500 μM arsenite (30 min). Scale bar, 10 μm. Profile intensity showing Aurora-A/Aurora-B/G3BP1 fluorescence signals of the white lines for each condition from the merged images.

M. Western blotting showed that the protein expression levels of Aurora-A, Aurora-B, and the SG core proteins G3BP1, TIA1 unchanged, whereas the phosphorylation level of eIF2α was increased in U251 cells with 100 μM arsenite (1 h), 500 μM arsenite (30 min).

N. Confocal imaging of non-target control (NTC) and Aurora-B shRNA1, shRNA2 treated with 100 μM sorafenib (2 h) , and 500 μM arsenite (30 min) to visualize the formation of SGs in U251 cells. Sorafenib did not induce SGs formation. Quantification of the proportion of cells with SGs formation (n=3) and the area of SGs per cell (n=50) after arsenite treatment. Mean ± SD, ns, non-significance.

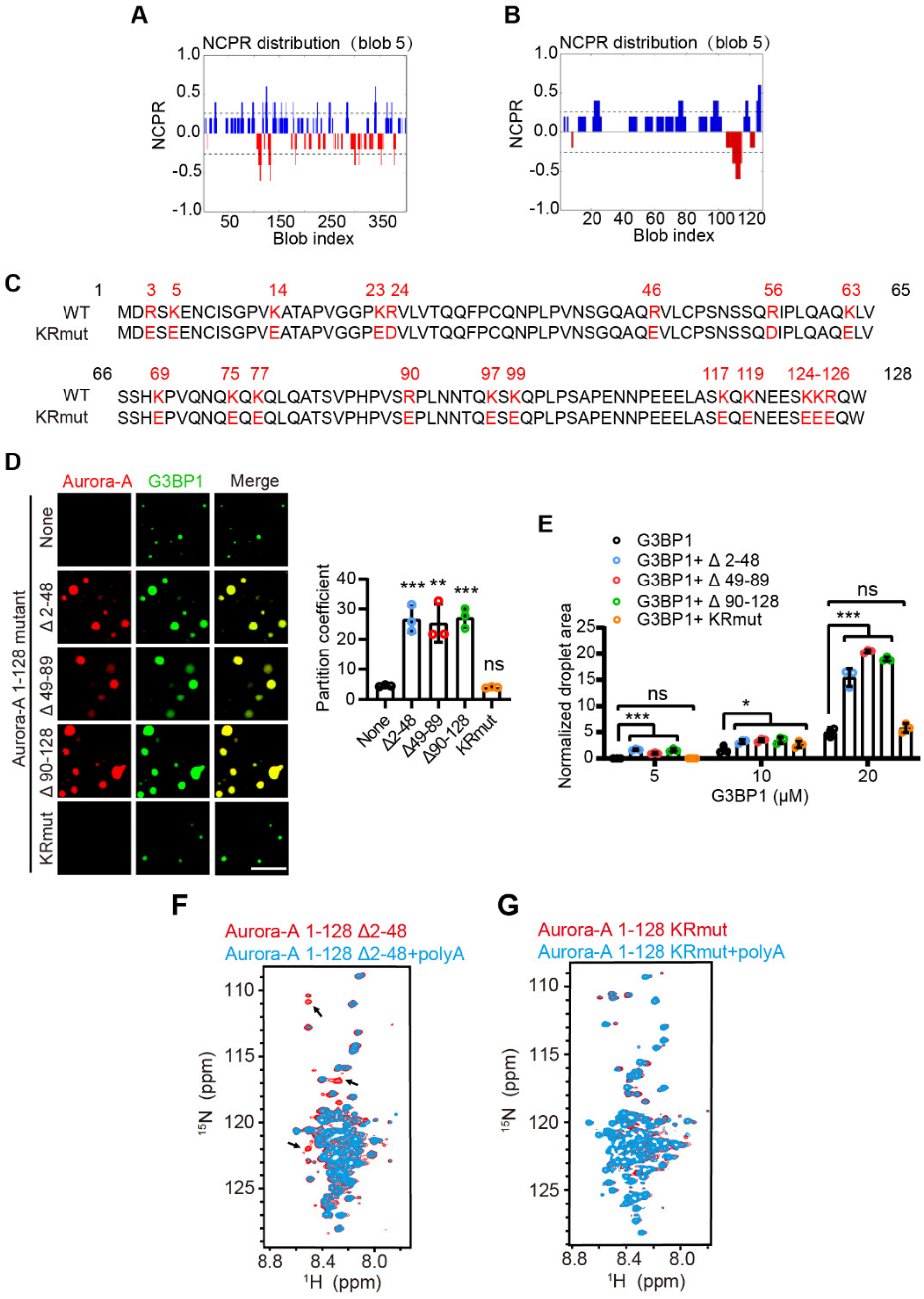
A
NCPR distribution（blob 5）
NCPR
Blob index
B
NCPR distribution（blob 5）
NCPR
Blob index
C
1 3 5 14 23 24 46 56 63 65
WT MDRSKENCISGPVKATAPVGGPKRVLVTQQFPCQNPLPVNSGQAQRVLCPSNSSQRIPLQAQKLV
KRmut MDESEENCISGPVEATAPVGGPEDVLVTQQFPCQNPLPVNSGQAQEVLCPSNSSQDIPLQAQELV
66 69 75 77 90 97 99 117 119 124-126 128
WT SSHKPVQNQKQKQLQATSVPHPVSRPLNNTQKSKQPLPSAPENNPEEELASKQKNEESKKRQW
KRmut SSHEPVQNQEQEQLQATSVPHPVSEPLNNTQESEQPLPSAPENNPEEELASEQENEESEEEQW
D
Aurora-A
G3BP1
Merge
Aurora-A 1-128 mutant
None
Δ 2-48
Δ 49-89
Δ 90-128
KRmut
Partition coefficient
None
Δ2-48
Δ49-89
Δ90-128
KRmut
***
**
***
ns
E
G3BP1
G3BP1+ Δ 2-48
G3BP1+ Δ 49-89
G3BP1+ Δ 90-128
G3BP1+ KRmut
Normalized droplet area
G3BP1 (μM)
ns
***
*
ns
***
F
Aurora-A 1-128 Δ2-48
Aurora-A 1-128 Δ2-48+polyA
15N (ppm)
1H (ppm)
G
Aurora-A 1-128 KRmut
Aurora-A 1-128 KRmut+polyA
15N (ppm)
1H (ppm)

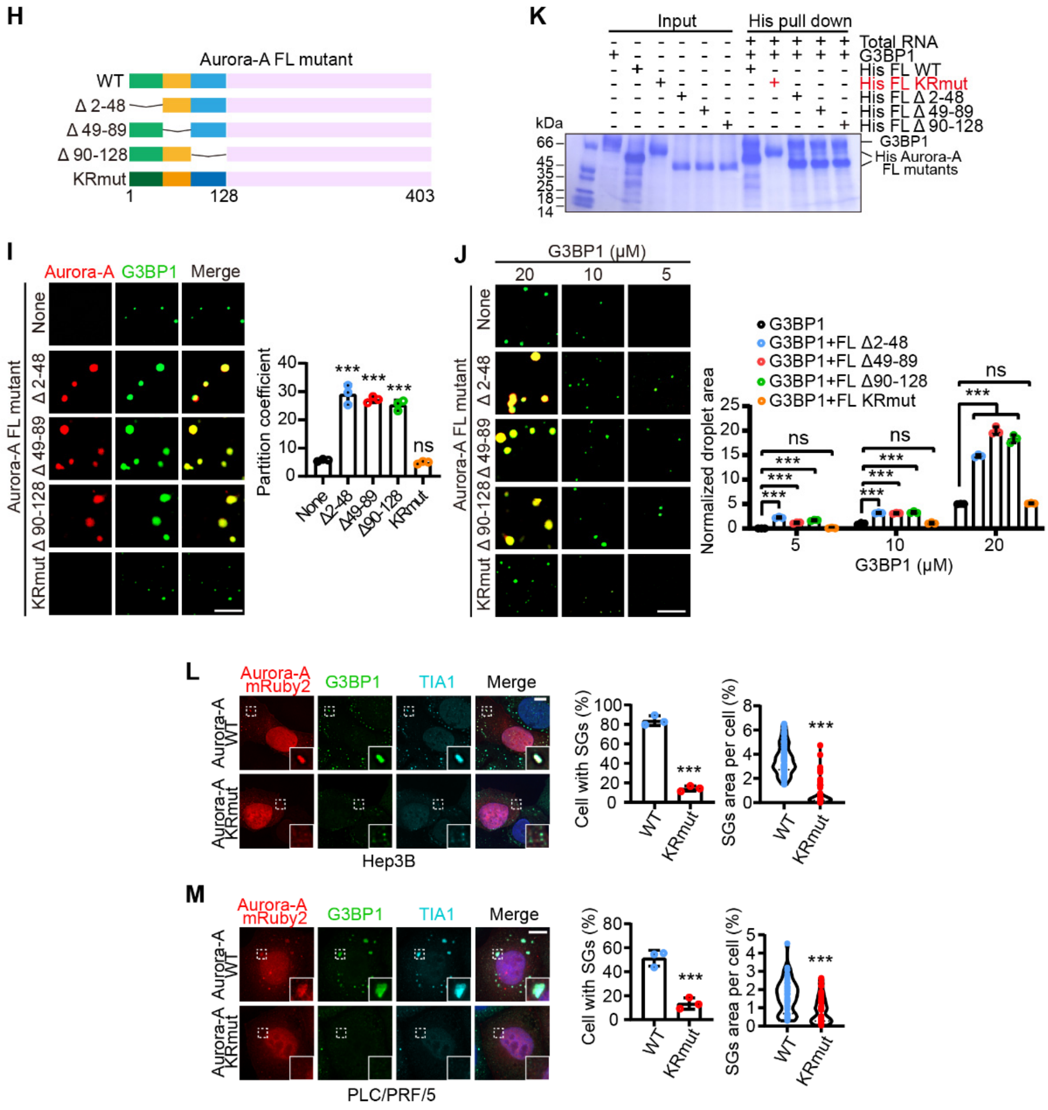

Figure S5. The KR amino acids in the IDR of Aurora-A interact with G3BP1-RNA.

A. The net charge per residue (NCPR) of Aurora-A full-length was analyzed using CIDER. The line at 0 (y axis) is the cutoff for the positive charge (>0, blue) and negative (<0, red) predictions.

B. The net charge per residue (NCPR) of Aurora-A IDR was analyzed using CIDER. The line at 0 (y axis) is the cutoff for the positive charge (>0, blue) and negative (<0, red) predictions.

C. Aurora-A IDR K/R residues mutant (KRmut) sequence and mutant sites (red).

D. Phase separation of purified recombinant proteins 1 μM Alexa Fluor™ 594-labeled Aurora-A IDR mutants or truncations (red) with 20 μM Alexa Fluor™ 488-labeled G3BP1 (green) in the presence of 50 ng/μL total RNA.. Images show representative data from three independent experiments. Scale bar, 10 μm. Quantification of the Aurora-A IDR mutants or truncations partition coefficient from three independent measurements. Mean ± SD, **p<0.01; ***p<0.001; ns, non-significance.

E. Quantification of the area of G3BP1 droplets in Fig. 5B. n=3, Mean ± SD, *p<0.05; ***p<0.001; ns, non-significance.

F. Overlay of ^1H-^{15}N-HSQCs panorama of either 100 μM Aurora-A 1-128 Δ2-48 alone (red) or in complex with a 1:1 ratio of 25nt-polyA (blue). Selected residues experiencing large chemical shift perturbations are labeled with arrows.

G. Overlay of ^1H-^{15}N-HSQCs panorama of either 100 μM Aurora-A 1-128 KRmut alone (red) or in complex with a 1:1 ratio of 25nt-polyA (blue).

H. Constructs used to investigate the function of individual truncated mutants of Aurora-A full-length (FL).

I. Phase separation of 1 μM Alexa Fluor™ 594-labeled Aurora-A FL mutants or truncations (red) with 20 μM Alexa Fluor™ 488-labeled G3BP1(green) in the presence of 50 ng/μL total RNA. The assay was performed using purified recombinant proteins in phosphate buffer (20 mM Na_2HPO_4, 150 mM NaCl, pH 7.0). Images show representative data from three independent experiments. Scale bar, 10 μm. Quantification of the Aurora-A FL mutants or truncations partition coefficient from three independent measurements. Mean ± SD, ***p<0.001; ns, non-significance.

J. LLPS of purified G3BP1 at different concentrations with 50 ng/μL total RNA, with 1 μM Aurora-A FL mutants or truncations respectively in phosphate buffer (20 mM Na_2HPO_4, 150 mM NaCl, pH 7.0). Images show representative data from three independent experiments. Scale bar, 10 μm. Quantification of the area of G3BP1 droplets. n=3, Mean ± SD, ***p<0.001; ns, non-significance.

K. His pull-down assay demonstrated RNA-dependent interaction between G3BP1 and the K/R residues of Aurora-A 1-128. Purified His-tagged Aurora-A FL mutants or truncations (200 μg) were incubated with untagged G3BP1 (200 μg) and total RNA (200 μg). Proteins bound to Ni-NTA beads were analyzed by Coomassie blue staining.

L. Confocal imaging of mRuby2-tagged Aurora-A-WT and Aurora-A-KRmut treated with 10 μM sorafenib (2 h) to visualize the formation of SGs in Hep-3B cells. Nucleus was stained with DAPI (blue). Scale bar, 10 μm. Quantification of the proportion of cells with SGs formation (n=3) and the area of SGs per cell (n=50), Mean ± SD, ***p<0.001.

M. Confocal imaging of mRuby2-tagged Aurora-A-WT and Aurora-A-KRmut treated with 25 μM sorafenib (2 h) to visualize the formation of SGs in PLC/PRF/5 cells. Nucleus was stained with DAPI (blue). Scale bar, 10 μm. Quantification of the proportion of cells with SGs formation (n=3) and the area of SGs per cell (n=50), Mean ± SD, ***p<0.001.

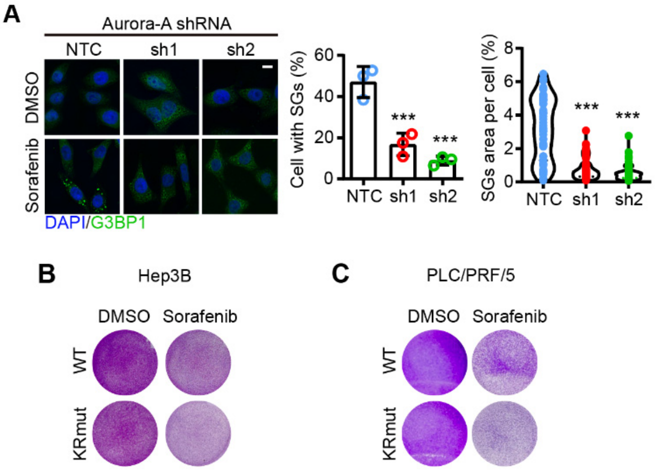

Figure S6. Aurora-A-orchestrated SGs formation drives sorafenib chemoresistance.

A. Confocal imaging of non-target control (NTC) and Aurora-A shRNA1, shRNA2 treated with 10 μM sorafenib (2 h) to visualize the formation of SGs. Quantification of the proportion of cells with SGs formation (n=3) and the area of SGs per cell (n=50). Mean ± SD, ***p<0.001

B. Crystal violet staining of Hep-3B Aurora-A WT or KRmut cell lines 2 d after treatment with DMSO or 10 μM sorafenib.

C. Crystal violet staining of PLC/PRF/5 Aurora-A WT or KRmut cell lines 2 d after treatment with DMSO or 25 μM sorafenib.

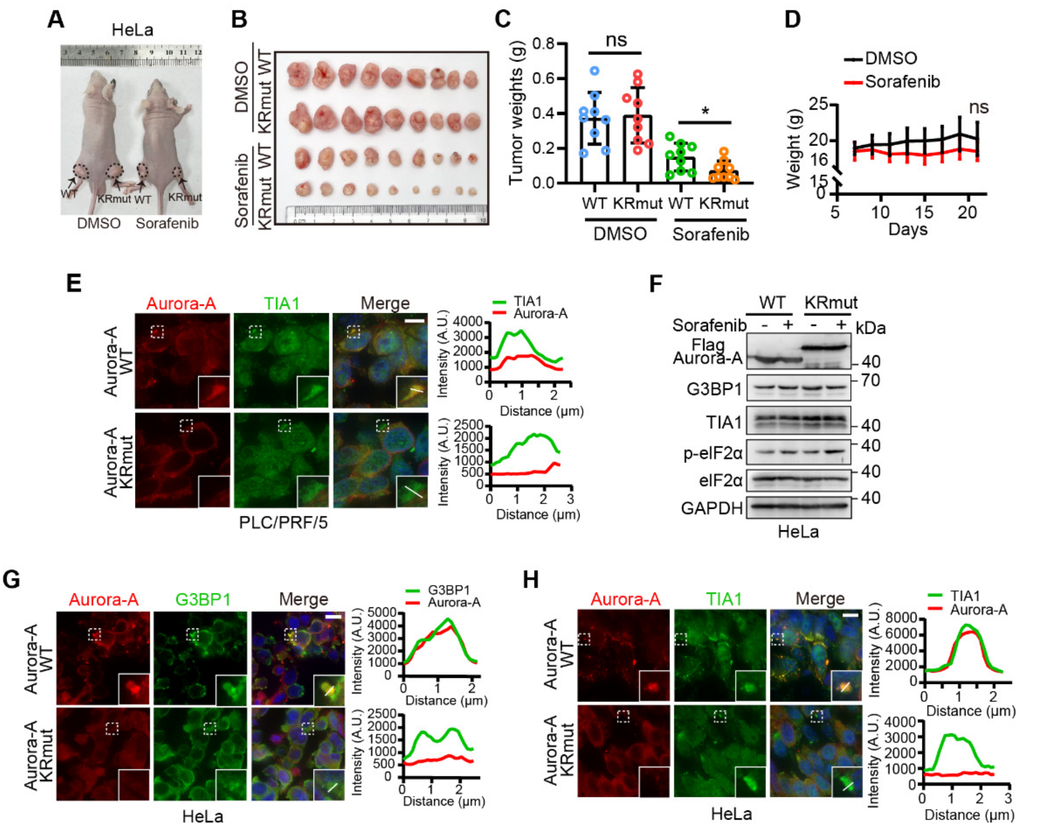

Figure S7. Aurora-A-mediated SGs formation promotes resistance to sorafenib in vivo.

A. Representative images of mice at 21 days post sorafenib administration. Sorafenib 30mg/kg, i.g.

B. Photographs of HeLa xenograft tumors excised from individual mice.

C. The weight of excised HeLa xenograft tumors at 21 days post sorafenib administration. n=9, Mean ± SD, *p<0.05; ns, non-significance.

D. The body weight of the mice was monitored for 21 days. n=10. ns, non-significance.

E. Immunofluorescence staining for TIA1 and Aurora-A (WT or KRmut) in PLC/PRF/5 xenograft tumors derived from fig. 7B.

F. Western blotting verified the expression of Aurora-A WT and KRmut in HeLa xenograft tumors from Fig. S7B. Sorafenib administration elevated eIF2α phosphorylation while leaving the total eIF2α protein level unchanged.

G. Immunofluorescence staining for TIA1 and Aurora-A (WT or KRmut) in HeLa xenograft tumors derived from Fig. S7B.

H. Immunofluorescence staining for G3BP1 and Aurora-A (WT or KRmut) in HeLa xenograft tumors derived from Fig. S7B.

Table S1. Detail information for cell lines and reagents used.

Cell lines	Company	Cat number
HeLa	CAS, Shanghai	TCHu187
PLC/PRF/5	ATCC	CRL-8024
Hep-3B	ATCC	HB-8064
HEK293T	ATCC	CRL-3216
**Reagent/resource**	**Reference or source**	**Cat number**
Sorafenib	Selleck	S7397
Sodium arsenite	Gift from Dr. Ke Ruan in USTC	N/A
Oxaliplatin	Selleck	S1224
5-Fluorouracil	Selleck	S1209
Bortezomib	Selleck	S1013
Osimertinib	Selleck	S7297
Rociletinib	Selleck	S7284
MLN8237	Selleck	S1133
Cycloheximide	Sigma	C1988
Puromycin	Sigma	P8833
G418	Sangon Biotech	A600958-0001
Doxycycline	MCE	HY-N0565B
SYTOX Green dye	Invitrogen	S7020
cOmplete His-Tag purification resin	Roche	COHISR-RO
Trizol	SuperfecTRI	3101-100
DAKO Fluorescence Mounting Medium	Dako North America	S302380-2
SteadyPure Universal RNA Extraction Kit	AG	AG21017

Table S2. Detail information for antibodies used.

Antibodies	Company	Cat number
Aurora-A for WB	Cell Signaling	4718
Aurora-A for IF	BD biosciences	BD610939
Aurora-B	BD biosciences	611082
BuGZ	Sigma	HPA017013
β-actin	Proteintech	66009-1-Ig
eIF2α	Cell Signaling	5324
Flag	Proteintech	66008-3-Ig
G3BP1	Proteintech	13057-2-AP
TIA1	Proteintech	12133-2-AP
GAPDH	Proteintech	60004-1-Ig

phospho Aurora kinase	Cell Signaling	2914
phospho Aurora A (Thr288)	Cell Signaling	3079
phospho eIF2α (Ser51)	Cell Signaling	3597
Ribosomal Protein S6	Santa Cruz	sc-74459
HRP-conjugated anti-rabbit secondary antibody	Jackson	111-035-003
HRP-conjugated anti-mouse secondary antibody	Jackson	115-035-003
Alexa 488 conjugated anti rabbit secondary antibody	Jackson	111-545-144
Alexa 594 conjugated anti mouse secondary antibody	Jackson	115-585-146

Table S3. Oligonucleotide sequence of shRNAs, sgRNAs, and primers for PCR.

**Genes**	**Sequences**
Aurora-A shRNA1	GCAAGCAGCCCCTGCCATCGG
Aurora-A shRNA2	CCTGTCTTACTGTCATTCGAA
Aurora-B shRNA1	CCTGCGTCTCTACAACTATT
Aurora-B shRNA2	GAAGAGCTGCACATTTGACG
G3BP1 sgRNA	AAGCCTAGTCCCCTGCTGGT
G3BP2 sgRNA	CAGTCCGCTGCTTGTAGGGC
Aurora-A shRNA2 resistance fw	AGAATTGGCAAATGCCCTATCATATTGCCACTCAAAGAGAGTTATTCAT
Aurora-A shRNA2 resistance rev	ATGAATAACTCTCTTTGAGTGGCAATATGATAGGGCATTTGCCAATTCT
Aurora-A fw	ATGGACCGATCTAAAGA
Aurora-A rev	CTAAGACTGTTTGCTAG
Aurora-A 1-128 rev	CTACCACTGCCTCTTTTTTGA
Aurora-A 129-403 fw	ATGGCTTTGGAAGACTTTGA
Aurora-A Δ2-48 fw	ATGTGTCCTTCAAATTCTTCCC
Aurora-A Δ49-89 fw	AGGCTCAGCGGGTCTTGAGGCCACTGAATAAC
Aurora-A Δ49-89 rev	GTTATTCAGTGGCCTCAAGACCCGCTGAGCCT
Aurora-A Δ90-128 fw	AGTGTACCTCATCCTGTCTCCGCTTTGGAAGACTTTG
Aurora-A Δ90-128 rev	CAAAGTCTTCCAAAGCGGAGACAGGATGAGGTACACT
Aurora-A 1-128 K,R to D,E	See Fig. S5C

References

1. Cheng A, Xu T, You W, Wang T, Zhang D, Guo H, et al. A mitotic NADPH upsurge promotes chromosome segregation and tumour progression in aneuploid cancer cells. Nat Metab. 2023;5(7):1141-58.
2. Yang P, Mathieu C, Kolaitis RM, Zhang P, Messing J, Yurtsever U, et al. G3BP1 Is a Tunable Switch that Triggers Phase Separation to Assemble Stress Granules. Cell. 2020;181(2):325-45 e28.
3. Lin Y, Protter DS, Rosen MK, Parker R. Formation and Maturation of Phase-Separated Liquid Droplets by RNA-Binding Proteins. Mol Cell. 2015;60(2):208-19.
4. Guillen-Boixet J, Kopach A, Holehouse AS, Wittmann S, Jahnel M, Schlussler R, et al. RNA-Induced Conformational Switching and Clustering of G3BP Drive Stress Granule Assembly by Condensation. Cell. 2020;181(2):346-61 e17.
5. Song D, Kuang L, Yang L, Wang L, Li H, Li X, et al. Yin and yang regulation of stress granules by Caprin-1. Proc Natl Acad Sci U S A. 2022;119(44):e2207975119.
6. Kovalski JR, Sarioglu G, Subramanyam V, Hernandez G, Rademaker G, Oses-Prieto JA, et al. Functional screen identifies RBM42 as a mediator of oncogenic mRNA translation specificity. Nature Cell Biology. 2025;27(3):518-29.
7. Dumas L, Shin S, Rigaud Q, Cargnello M, Hernández-Suárez B, Herviou P, et al. RNA G-quadruplexes control mitochondria-localized mRNA translation and energy metabolism. Nature Communications. 2025;16(1).
8. Mateusz Szczerba BJ, Francesco Acciai, Carolina Gogerty, Megan McCaughan, Jacqueline Williams, Karen V. Kibler, Bertram L. Jacobs. Canonical cellular stress granules are required for arsenite-induced necroptosis mediated by Z-DNA–binding protein 1. science signaling. 2023;16(776).
9. Fujikawa D, Nakamura T, Yoshioka D, Li Z, Moriizumi H, Taguchi M, et al. Stress granule formation inhibits stress-induced apoptosis by selectively sequestering executioner caspases. Curr Biol. 2023;33(10):1967-81 e8.
